# Role of particle size and element characteristics on shear response at sandy soil-textured surface interfaces

**DOI:** 10.1038/s41598-025-26385-3

**Published:** 2025-11-27

**Authors:** Mu’ath I. Abu Qamar, Azhar G. Hamad, Ammar A. Alshannaq, Mohammad F. Tamimi, Motasem I. Al-Qura’an

**Affiliations:** 1 Department of Civil Engineering, Yarmouk University, Irbid, 21163 Jordan; 2 Department of Civil Engineering, University of Thi-Qar, Thi-Qar, 64001 Iraq; 3 AQ Saray Engineering, Amman, Jordan

**Keywords:** Structured rough surfaces, Sand, Roughness, Particle size, Interface shear testing, Engineering, Civil engineering

## Abstract

A fundamental understanding of shear behavior at the interface between foundation elements with innovative surfaces that include bio-inspired or structured element designs is critical in the design of many geotechnical structures. Some geotechnical applications could benefit from the use of surfaces with structured roughness form that mobilize larger shear resistances than conventional interfaces with random roughness form. However, several parameters such as soil properties, particle size, surface roughness, geometry of surface elements may influence the shear behavior at the interface between soil and surfaces with structured roughness which requires further research. To study the possible effects of these parameters on shear resistance, a series of interface direct shear tests were performed for 7 sands with varying particle sizes and four aluminum surfaces (three textured with trapezoidal-like elements and a smooth surface). When the 7 sands sheared against the same textured surface, the shear resistance decreases with increasing element height to mean particle diameter ratio (*h*/*D*_50_) and increases with increasing element-to-element spacing to height ratio (*S*/*h*). A parametric study on the geometrical characteristics of surface elements and soil particle size revealed that the *h*/*D*_50_ and *S*/*h* ratios could quantitatively capture the interface load-transfer mechanisms between sand and textured surfaces. Based on the results obtained, it was found that the effect of particle size of test sand on shear resistance diminishes as *h*/*D*_50_ and *S*/*h* decreases (i.e., surfaces with closely spaced elements). It was also found that, for identical surface characteristics, particle size affects the mobilized resistance: higher shear strengths are achieved when the sand’s mean particle diameter (*D*_50_) closely matches the asperity (element) height of the textured surface.

## Introduction

A fundamental understanding of shear behavior at the interface between foundation elements with innovative surfaces that include bio-inspired or structured element designs is critical in the design of many geotechnical structures. The design and overall stability of foundation elements utilizing structured roughness form are significantly influenced by load-transfer mechanisms that occur at the soil-foundation interface under static and/or cyclic shearing loading. However, parameters derived from soil and surface characteristics which may control the shear load-transfer mechanisms and the shear strength at the interface between surfaces with structured roughness form and cohesionless (sandy) soils are not fully understood. The study of the effect of these parameters could greatly enhance the design of foundation elements with structured roughness form for optimal load-carrying capacity. The shearing behavior at the interface between construction materials and cohesionless and cohesive soils has been investigated through conducting interface shear tests utilizing various shear testing configurations as follows: (1) interface simple shear^1,2^; (2) interface ring shear^3^; (3) interface direct shear^4–10^; (4) axisymmetric interface shear^11,12^; and (5) interface borehole shear^13–16^. Despite of the known limitations of these test setups, the body of experimental work greatly enhanced the understanding of the effects of specific parameters that significantly affect the shear resistance and interface response such as surfaces roughness level and form, particle size and morphology, soil density, soil gradation, and stress history^2,6,12,13,16–23^. Research studies have investigated the directionally dependent response of snakeskin-inspired surfaces^24–26^ and surfaces with structured roughness^17,18^ in the terms of interface shear resistance and soil volume change due to their asymmetric roughness form. The interfaces with asymmetric roughness mobilize greater interface shear resistance and soil dilation in one direction (i.e., against the asperities’ sharp edge or right-angle side) than in the opposite direction (i.e., along the asperities’ milder slope or inclined side), The difference in resistance is known as aeolotropy, anisotropy, or frictional directionality. It is widely accepted that the use of such surfaces could be beneficial for applications where larger resistance in one direction and smaller resistance in the opposite direction is desired. The influence of loading direction on interface shear resistance was not examined in the present study.

An extensive body of research has evaluated the influence of soil, geomaterial properties, and load-transfer mechanisms on interface shear response and behavior through performing interface shear tests^20^, . Factors such as test method, strain rate, and soil properties including initial soil structure and coefficient of uniformity have a lesser effect on the peak and residual interface shear strength than other factors including normal stress, particle angularity and morphology, mean particle diameter size (*D*_50_) and surface roughness^27^.

Potyondy^28^ and Brumund and Leonards^29^ were the first to identify that geomaterial surface roughness, soil moisture, particle size, particle angularity, particle mineralogy, as well as normal stress have a significant effect on interface shear strength. Brumund and Leonards^29^ further concluded that interface shear resistance increases with surface roughness, however, it has a limiting value equal to the internal shear resistance of sand. As the roughness increases, the load-transfer mechanism shifts shearing from a soil-surface interface into a soil-soil condition. However, it was not possible to develop a quantitative relationship between surface roughness and interface shear strength due to the lack of automated profiling devices.

**Fig. 1:**
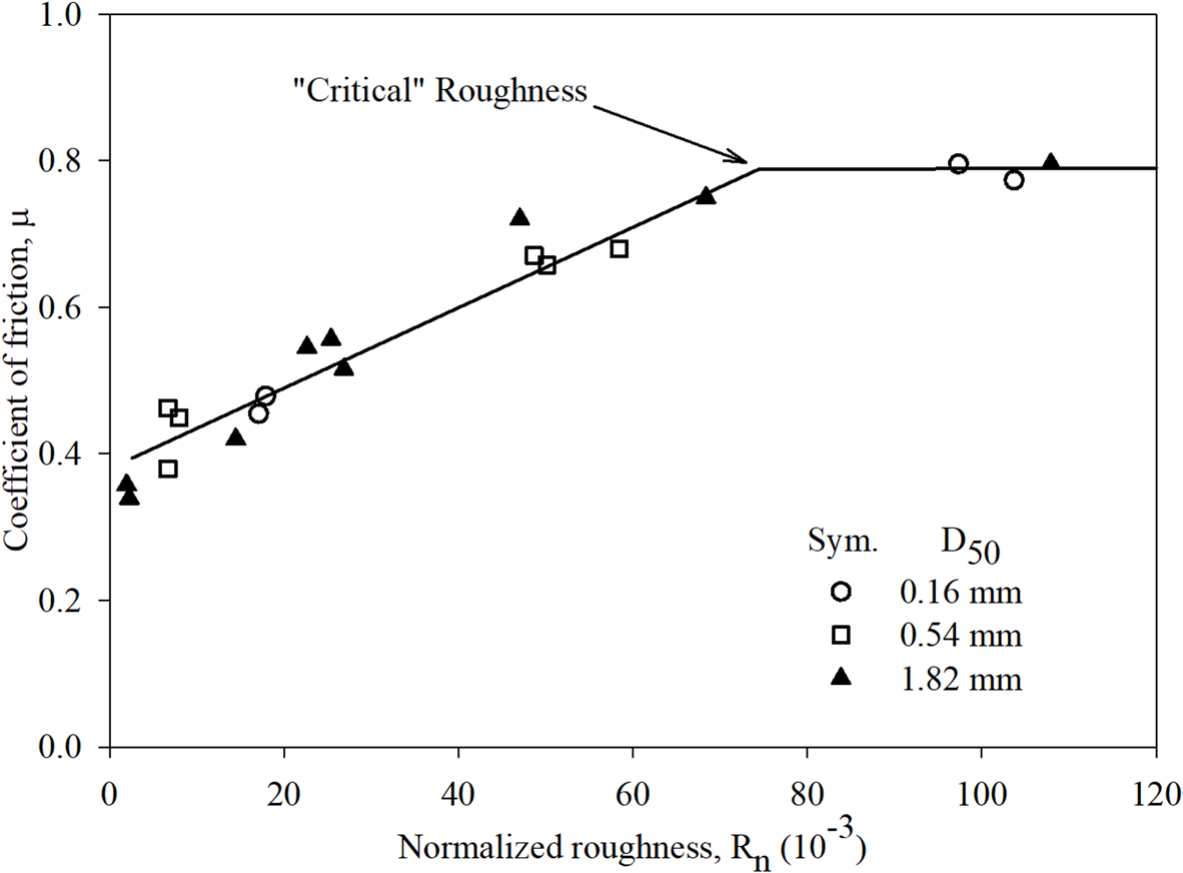
Relationship between coefficient of friction and surface roughness for sand-steel interfaces (modified after Uesugi and Kishida^30^).

The role of geomaterial surface roughness on interface shear resistance was initially investigated by Uesugi and Kishida^2,30^ through utilizing a modified simple shear apparatus to conduct tests on sand-steel interfaces. The behavior initially observed by Brumund and Leonards^29^ was quantitively measured by Uesugi and Kishida^2^ from a series of tests performed on a series of sands tested against rough steel plates. Based on results obtained from these tests, Uesugi and Kishida^2^ proposed the normalized roughness parameter [*R*_*n*_ = *R*_max_ (*L* = *D*_50_) / *D*_50_], which is defined as maximum roughness over a gauge length equal to the mean particle diameter (*D*_50_). A bilinear relationship (see Fig. 1) between surface roughness and interface friction was obtained when *R*_*n*_ is used to represent roughness of geomaterials.

The bilinear relationship consists of two portions namely left- and right-hand portions meets at a critical roughness of 75 × 10^− 3^. The interface friction increases as the *R*_*n*_ increases with load-transfer occurs through particles sliding at the surface. As the *R*_*n*_ approaches the critical roughness, the load-transfer occurs mainly within soil body and interface friction strength become equal to internal friction strength (or internal friction angle) under confinement. Further increase in surface roughness beyond the critical roughness resulted in no increase in interface friction angle or interface frictional strength (i.e., right-hand portion of the bilinear relationship).

The soil–geostructure interface resists applied shear loading by friction, passive resistance, or a combination of both, depending on the surface roughness^31,32^. Surfaces exhibiting very low or no roughness (i.e., smooth) resist applied loads primarily through friction, whereas surfaces with structured roughness form mobilize combined interface resistance incorporating both frictional forces and passive mechanisms such as local shear, particle displacement, soil entrapment, and passive wedge formation (or passive resistance)^11,12,22,31^. The passive resistance is defined as the soil resistance mobilized against bearing (or transverse) surface elements such as structured roughness asperities (i.e., a 90-degree surface element) positioned normal to the direction of relative displacement at the interface between soil and geostructure surface^32^.

For surfaces with structured roughness form, the measured total resistance is a combination of two components: (1) the frictional resistance component occurs along the untextured (smooth) contact areas through sliding at the soil-surface interface, and (2) the passive resistance component is mobilized by passive soil wedges and arises from local shear and particle displacement around textured surface elements. The variation in spacing between surface elements varies the relative contribution of interface friction and passive resistances to the total measured resistance. Therefore, the magnitude of interface passive resistance is not expected to remain constant across different element spacing^31,32^.

**Fig. 2:**
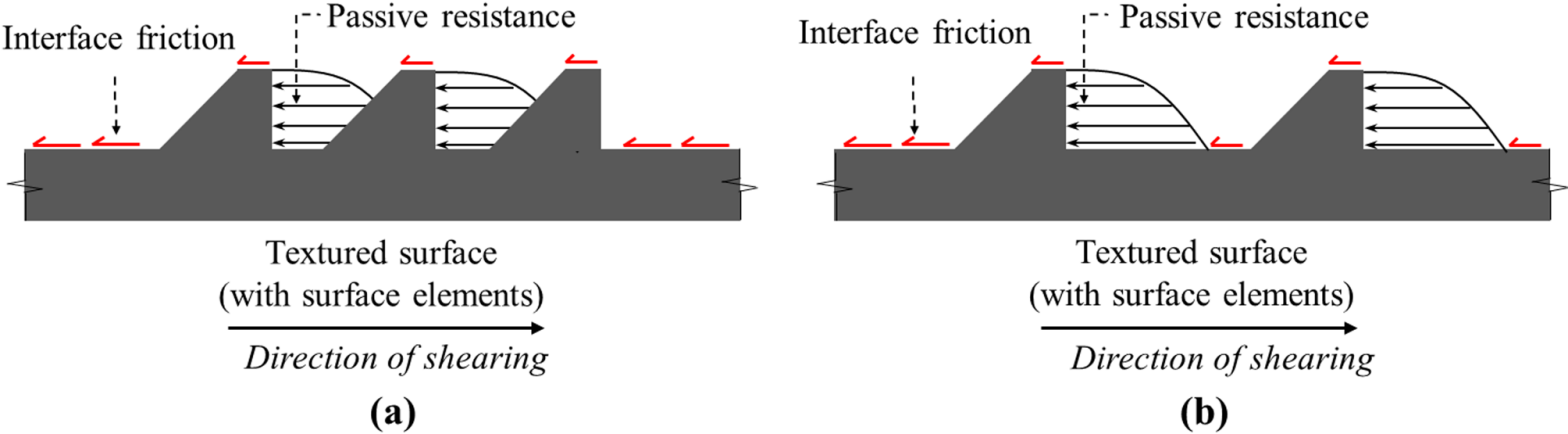
Shear load-transfer mechanisms at the interface between soil and textured surfaces during shearing: (**a**) partial passive wedges (spacing smaller than optimum) and (**b**) full passive wedges (optimum spacing).

For sandy soil, researchers have reported that the contribution of this additional resistance (named passive resistance) is controlled by the spacing between surface elements^31,33^. Irsyam and Hryciw^31^ and Qian et al.^35^ reported that the maximum passive resistance occurs when the spacing required to develop full passive wedges is achieved (i.e., optimum spacing) and that the applied load is transferred mainly through interface friction (i.e., contribution of passive resistance decrease) for surface elements with spacing smaller or larger than optimum spacing. The contribution of the additional (or passive) resistance to the total resistance is controlled by the geometry and spacing of surface elements. Figure 2a illustrates the formation of a partial passive wedge at the face of a rib in the case of closely spaced elements (smaller than optimum), while Fig. 2b shows the development of full passive wedges, corresponding to an optimum spacing configuration. As the spacing between surface elements increases, the number of passive wedges formed per unit length of contact decreases, thereby reducing their contribution to the total interface shear resistance.

The bilinear relationship observed by Uesugi and Kishida^30^, shown in Fig. 1, has been verified for surfaces with random roughness form including geomembranes, steel, concrete, fiber reinforced polymers (FRP), and wood^34,35^. However, the applicability of the bilinear relationship between interface shear strength (friction) and the roughness for structured surfaces in sandy soils has not been explored or experimentally investigated.

To better understand the coupled effect of particle size and structured roughness on the interface shear behavior of sand and textured surfaces, a series of monotonic interface shear tests were conducted utilizing a modified direct shear box. This paper presents results of interface shear tests on sand tested against textured surfaces with structured roughness in which test sand particle size as well as surface roughness characteristics were varied. Three textured surfaces with trapezoidal-like elements of different numbers and spacing having the same height, and a smooth (untextured) surface were used in all interface tests.

## Materials and experimental setup

### Test sands

**Fig. 3:**
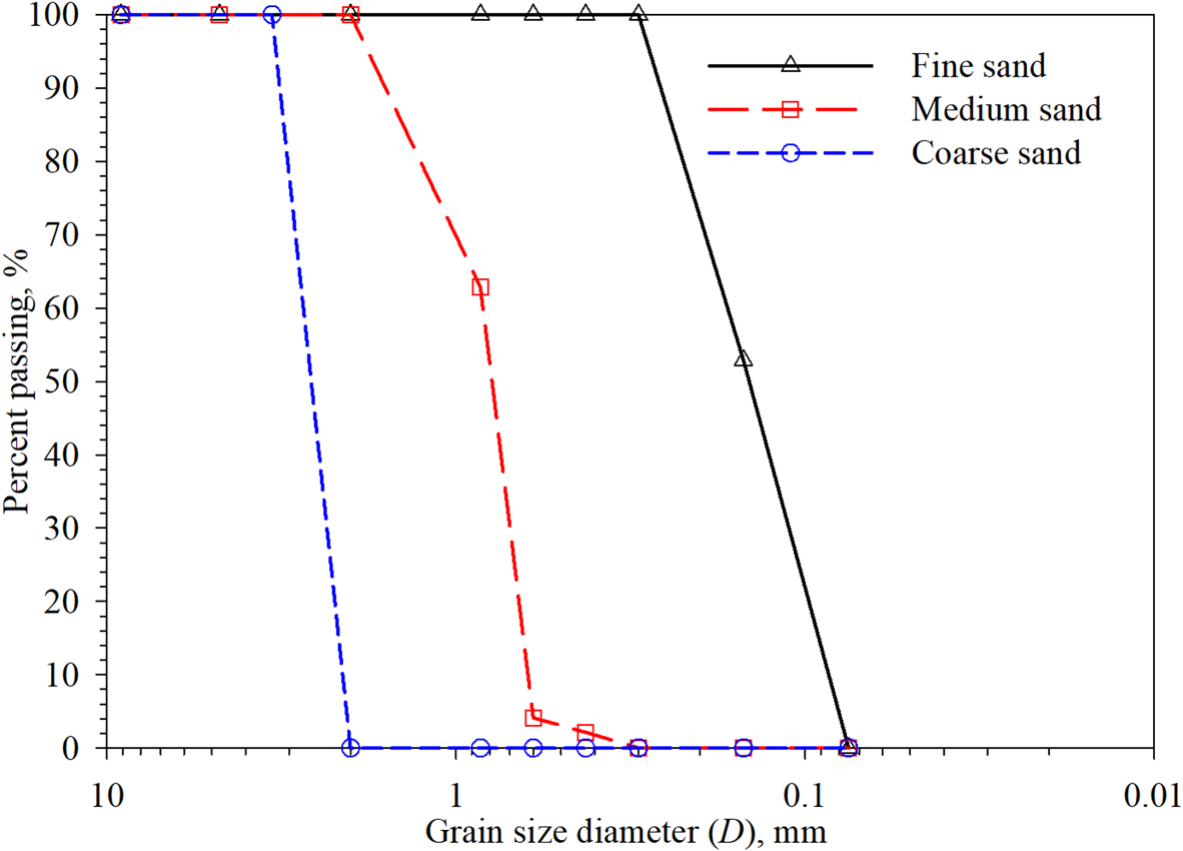
Grain-size distrbution curves of fine sand, medium sand, and coarse sand.

Seven different silica sands were tested in the present study. These sands are as follows: (1) Sand 1 or fine sand that consists of 50% sand passed through sieve#100 (opening size of 0.150 mm) and 50% sand retained on sieve #200 (opening size of 0.075 mm), the mean particle diameter (*D*_50_) of fine sand is 0.146 mm, (2) Sand 2 that consists of 50% sand passed through sieve #50 (opening size of 0.300 mm) and 50% sand retained on sieve #50 (opening size of 0.150 mm), the mixture has a mean particle diameter (*D*_50_) of 0.271 mm, (3) Sand 3 which comprises of 50% of Sand 1 and 50% of sand passed through sieve #20 (opening size of 0.850 mm) and retained on sieve #30 (opening size of 0.600 mm) (mixed by weight), Sand 3 has a mean particle diameter (*D*_50_) of 0.370 mm, (4) Sand 4 or medium sand which is sand passed through sieve #20 (opening size of 0.850 mm) and retained on sieve #30 (opening size of 0.600 mm) (mixed by weight), the particle mean diameter (*D*_50_) is 0.780 mm, (5) Sand 5 is a mixture of 50% sand 1 and sand that is 50% passed through sieve #8 (opening size of 2.360 mm) and retained on sieve #10 (opening size of 2.000 mm) (mixed by weight), the mean particle diameter (*D*_50_) of the mixture is 1.050 mm, (6) Sand 6 is a mixture of Sand 4 and sand that is 50% passed through sieve #8 (opening size of 2.360 mm) and retained on sieve #10 (opening size of 2.000 mm) (mixed by weight), the mean particle diameter (*D*_50_) of the mixture is 1.050 mm, and (7) Sand 7 or coarse sand which is sand passed through sieve #6 (opening size of 3.360 mm) and retained on sieve #10 (opening size of 2.00 mm), the particle mean diameter (*D*_50_) is 2.260 mm. According to the Unified Soil Classification System (USCS)^36^, the fine sand (labeled as Sand 1) is classified as poorly-graded (SP) sand with coefficient of uniformity (*C*_*u*_) *= D*_60_*/D*_10_ and coefficient of uniformity (*C*_*c*_) = (*D*_30_)^2^/(*D*_10_ × *D*_60_) of 1.96 and 0.79, respectively. Sand 4 (or the medium sand) is classified as poorly-graded (SP) sand with *C*_*u*_ and *C*_*c*_ values of 1.31 and 1.01, respectively. The *C*_*u*_ and *C*_*c*_ values for coarse sand (labeled as Sand 7), which is classified as poorly-graded (SP) are 1.12 and 0.94, respectively. The sands 2, 3, 5, 6 were extracted fine, medium, and coarse sand to produce sand with uniform particle sizes (range of particle sizes). The mean particle diameter *D*_50_ of these sand (i.e., 1 to 7) ranges from 0.146 to 2.26 mm. The grain-size distribution curves for the fine, medium, and coarse sands are shown in Fig. 3. It is important to note that the influence of test sands morphology on interface response to shear loading was not included in the present study. The test sands used herein were used to study the effect of particle size (i.e., *D*_50_) on the sand-structured surfaces interfaces shearing behavior. The basic properties of the test sands include soil solid’s specific gravity (*G*_s_), maximum void ratio (*e*_max_), and minimum void ratio (*e*_min_) are summarized in Table 1.

**Table 1:** Basic characteristics of sands used in the present study

Sand	*D*_max_ (mm)	*D*_min_ (mm)	*D*_50_ (mm)	*e*_max_	*e*_min_	*G*_s_	*ϕ* (°)
Sand 1	0.150	0.075	0.146	0.779	0.524	2.63	31.7
Sand 2	0.300	0.150	0.271	0.769	0.519	2.64	31.5
Sand 3	0.600	0.075	0.370	0.706	0.527	2.61	34.5
Sand 4	0.850	0.600	0.780	0.755	0.555	2.64	33.6
Sand 5	2.000	0.075	1.050	0.701	0.543	2.62	37.1
Sand 6	2.000	0.850	1.480	0.837	0.600	2.61	35.4
Sand 7	3.360	2.000	2.260	0.812	0.653	2.63	38.1

^†^Internal angle of friction (*ϕ*) – (°); *Specific gravity (*G*_s_).

### Shear test setup

**Fig. 4:**
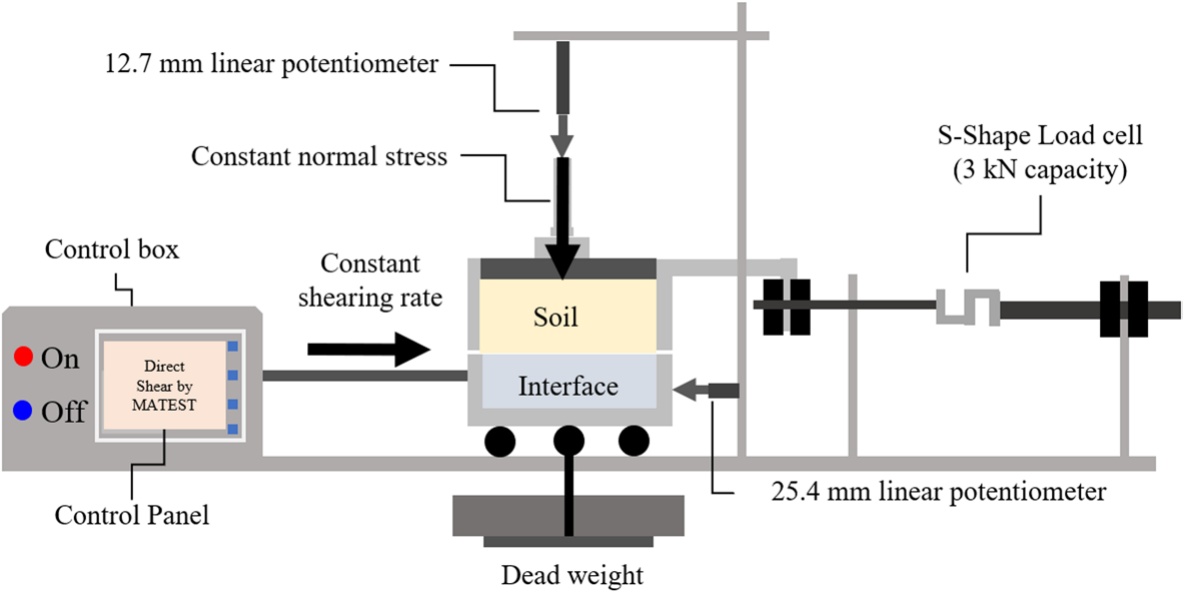
Components of the conventional direct or modified interface shear test system used in the study.

The internal angle of friction (*ϕ*) of the test sands were determined using the Sheartronic digital direct shear machine (S277-1 model, Matest, Italy). Figure 4 shows the components of the testing apparatus used to perform conventional direct or interface shear tests. The conventional apparatus was modified by replacing the bottom half of the shear box (See Fig. 4) with aluminum blocks of different surface conditions.

**Fig. 5:**
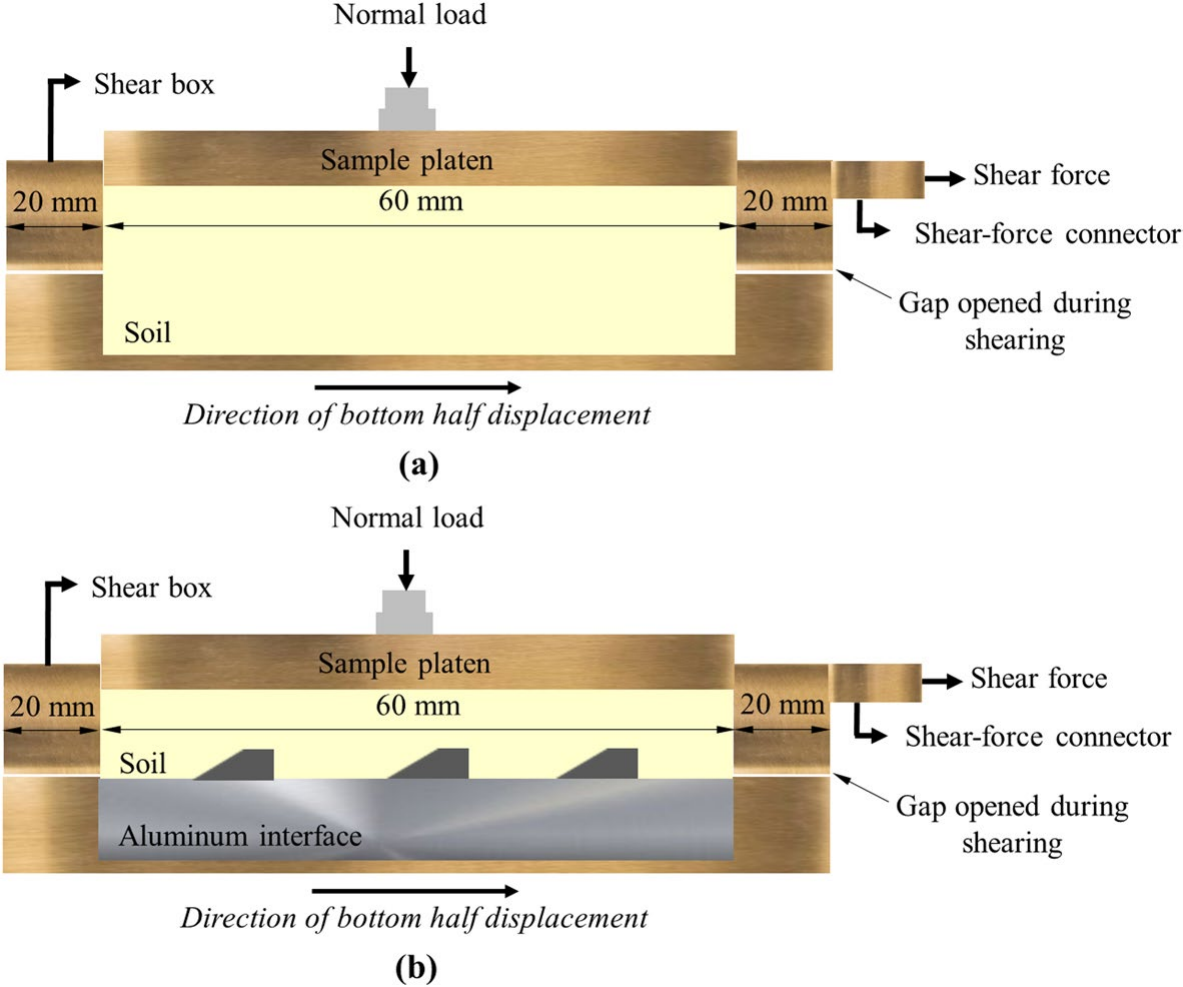
Shear test setup: (**a**) conventional shear box; and (**b**) modified interface shear box.

Figure 5a depicts the configuration of the conventional direct shear apparatus to test soil-soil condition and obtain the internal angle of friction of test sands. The square soil sample had a side length along the failure plane of 60 mm, which is about 20 times the maximum particle diameter (i.e., *D*_max_ of 3.00 mm for Sand 7 or coarse sand). A ratio of 10 times the maximum particle diameter is recommended by ASTM D3080/D3080M standard^37^ as the minimum sample size. Following the application of normal load (or normal stress) on the top of the sand specimen and prior to shearing, a gap with thickness equal to the diameter of the largest sand particle in the specimen was created between the bottom and top halves of the shear box^37^. The target gap thickness was achieved through rotating two screws fitted in the corners of the top half of the shear box shown in Fig. 5a. The shearing was initiated after removing the screws. During shearing, the bottom half of the shear box was displaced horizontally at constant rate of 0.0083 mm/s (or 0.5 mm/min) while top shear box was maintained stationary. The bottom half was moved to a total horizontal (shear) displacement of 10 mm. All sand specimens (Sand 1 to 7) were prepared at relative density (*D*_r_) of 80 ± 1% in all conventional and interface direct shear tests.

Figure 5b illustrates the configuration of the modified shear box that was utilized to determine the shear resistance at the interface between sand and structured aluminum surfaces with different characteristics (i.e., roughness, element or asperity spacing, number of elements or asperities per contact length). During the preliminary phase of the study, the interface specimens were designed to exactly occupy the volume of the bottom half of the shear box. The external dimensions of the bottom shear box are 100 mm × 100 mm × 20 mm, and the dimensions of the interface materials are 60 mm × 60 mm × 20 mm. The 20 mm margins on each side of the bottom half, along the shearing direction (see Fig. 5), function as an extended untextured interface during shearing. In this case, the size of the aluminum surface is larger than that of the size of soil sample to prevent soil leakage and ensure a constant contact area between the soil sample and the surface in a certain range of shear displacement. This configuration was adopted from previous studies^24–26,38^. The original bottom half of the shear box was used to accommodate aluminum blocks with different surface conditions. Similar to conventional direct shear tests, the bottom half of the shear box was moved horizontally at a constant shearing rate of 0.0083 mm/s (or 0.5 mm/min) to a total horizontal (shear) displacement of 10 mm relative to the stationary top half of the shear box. The soil-interface material shear setup used herein is known as the lower-plate setup where the interface materials are placed first then the soil is being filled over the interface prior to the application of normal stress and moving the bottom half horizontally. In the upper-plate shear setup, which is the other commonly used setup, the soil specimen is initially filled in the stationary bottom half of the shear box followed by moving the top half shear box horizontally at a constant rate^7^. Ho et al.^3^ examined the influence of the soil-interface material shear setup on the critical-state interface friction angle utilizing a ring shear device. It was observed that the critical-state friction angle obtained from upper-plate setup was lower than the angle from lower-plate shear setup. In the present study, the lower-plate shear setup is adopted based on previous research studies conducted to evaluate the interface shear behavior between sand and interface materials^7,13,18,19,39^.

### Interface roughness design

**Fig. 6:**
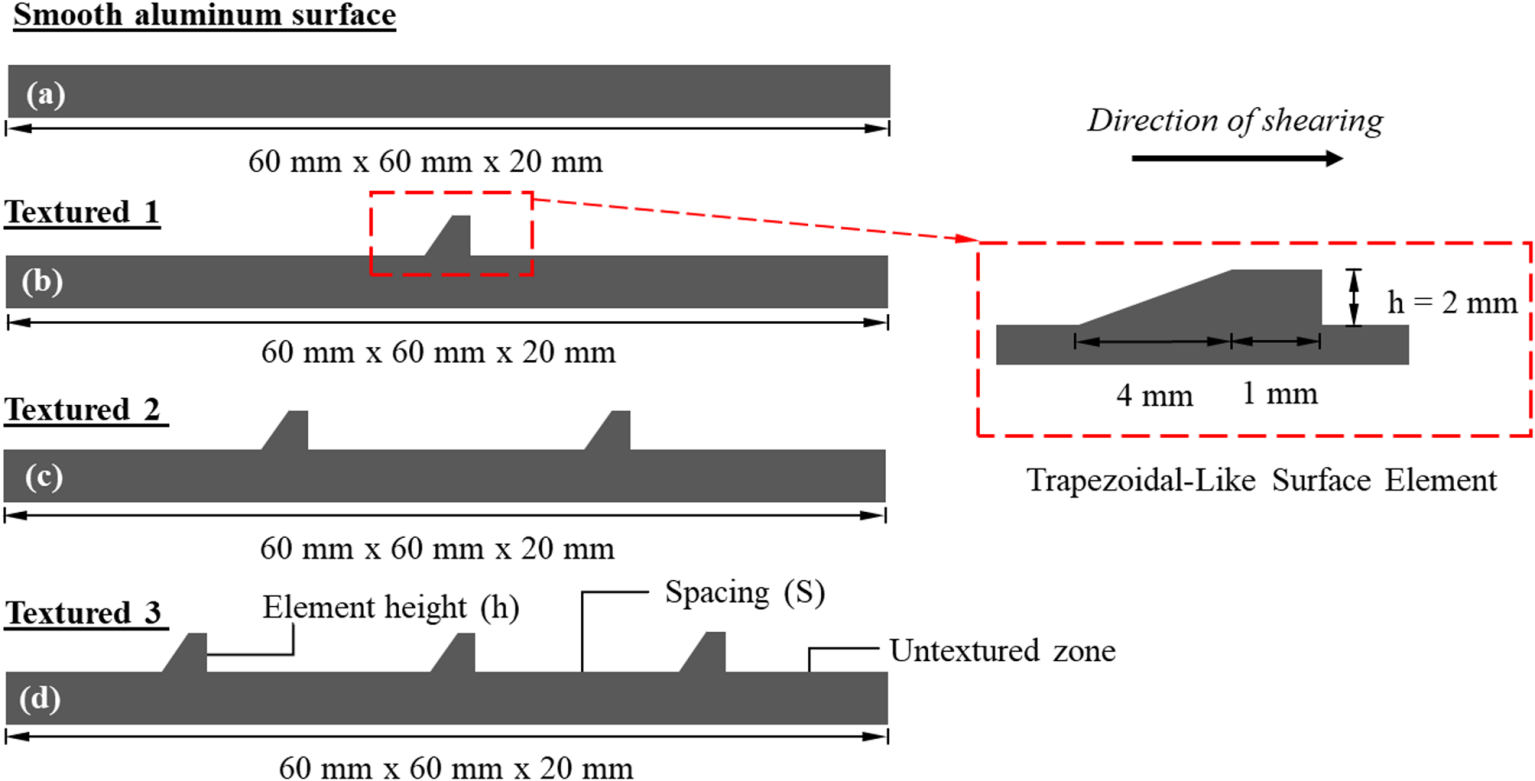
Design and geometry of interfaces used in this study: (**a**) smooth (untextured); (**b**) textured 1 (one element); (**c**) textured 2 (two elements); (**d**) textured 3 (three elements).

It is widely accepted that larger interface resistance of foundation elements under shear loading can be achieved if surfaces with structured elements or asperities are used. In order to simulate the surface of conventional and innovative foundation elements, four different surfaces made with aluminum were used in the present study. These surfaces are as follows: (1) smooth with no surface elements, (2) rough with one trapezoidal-like element (labeled as Textured 1), (3) rough with two trapezoidal-like element (labeled as Textured 2), and (4) rough with three trapezoidal-like element (labeled as Textured 3). Figure 6a-d shows the surface condition and dimensions of the test interface materials (i.e., smooth and textured). The topography (i.e., the element shape, element pattern, and element spacing) were described using the asperity height (*h*), the asperity spacing (*S*) and a constant asperity base length. The physical meaning of *h* and *S* are illustrated in Fig. 6d. Several roughness parameters such as centerline average roughness (*R*_*a*_), maximum vertical peak-to-valley roughness (*R*_*t*_) measured over the entire travel length, and maximum vertical peak-to-valley distance (*R*_max_) measured over a certain gauge length or a length over the mean particle size (*D*_50_) have been used to quantify the roughness of random rough surface by previous studies in the literature^2,40^. It is well known that roughness of surface over the contact area at the interface with soil plays a crucial role in the behavior under shear loading. Another well-known roughness parameter (i.e., the normalized roughness parameter (*R*_*n* =_ *R*_max_ / *D*_50_)) that was first introduced by Uesugi and Kishida^2^ has been widely adopted by other researchers^7,12,41,42^. The normalized roughness (*R*_*n*_) indicates the relative value of surface roughness to the sand particle size, the *R*_*n*_ (for same *R*_max_) increases as the particle size of sand grains decreases. As concluded by several research studies^7,12,41,42^, higher values of *R*_*n*_ lead to enhanced interlocking at the interface, which results in increasing the interface shear strength. As the surfaces used in the present study were machined to produce contact area with sand that consist of smooth (untextured areas) and trapezoidal-like elements, therefore, it is more meaningful in this context if the parameter used to quantify roughness (i.e., height of surface element or asperity) is observed over the entire length related to sand particle size; accordingly, *h/D*_50_ is used in the results presented in this paper. Tests were performed against surfaces with asperities of variable spacing (*S*) and constant height (*h*) in sands of varying particle sizes (i.e., varying *D*_50_) on the interface strength. Untextured regions approximately 10 mm in length were created on both ends of the textured section to reduce boundary effects, adopting the approach used by previous researchers^4,15,43–46^.

Table 2 summarizes the characteristics of the smooth and textured interfaces used in the present study. It is important to highlight that the asperity height (*h*) is not directly comparable to the maximum roughness (*R*_max_) defined by Kishida and Uesugi^30^, as asperity height (*h*) represents the entire surface, whereas *R*_max_ is measured over a length equivalent to the mean particle diameter (*D*_50_) of the soil grains. Asperity height parameter, termed as *h* or *R*_*t*_, that is based on the entire surface profile has been widely adopted by other researchers^17,20,47–53^.

**Table 2:** Characteristics of the interfaces used in the interface shear tests.

Surface no.	No. of elements, *N*	Element height, h (mm)	Spacing between elements, S (mm)	Element characteristic ratio, S/h
Smooth (Untextured)	0	2	0	0
Textured 1	1		55	27.5
Textured 2	2		25	12.5
Textured 3	3		15	7.5

## Test results and discussion

### Test results

**Fig. 7:**
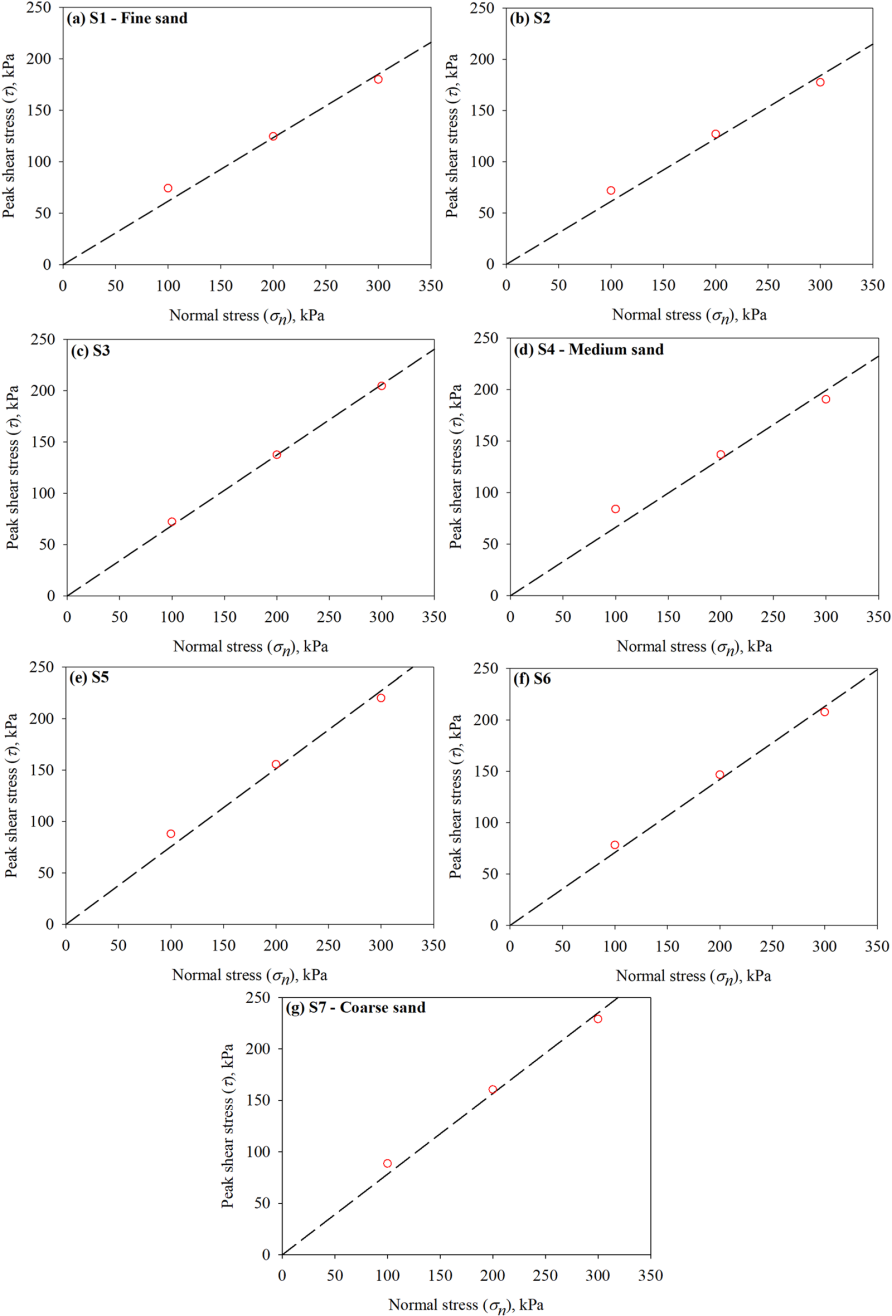
Resutls from conventional direct shear tests conducted on all sands (**a–g**).

**Fig. 8:**
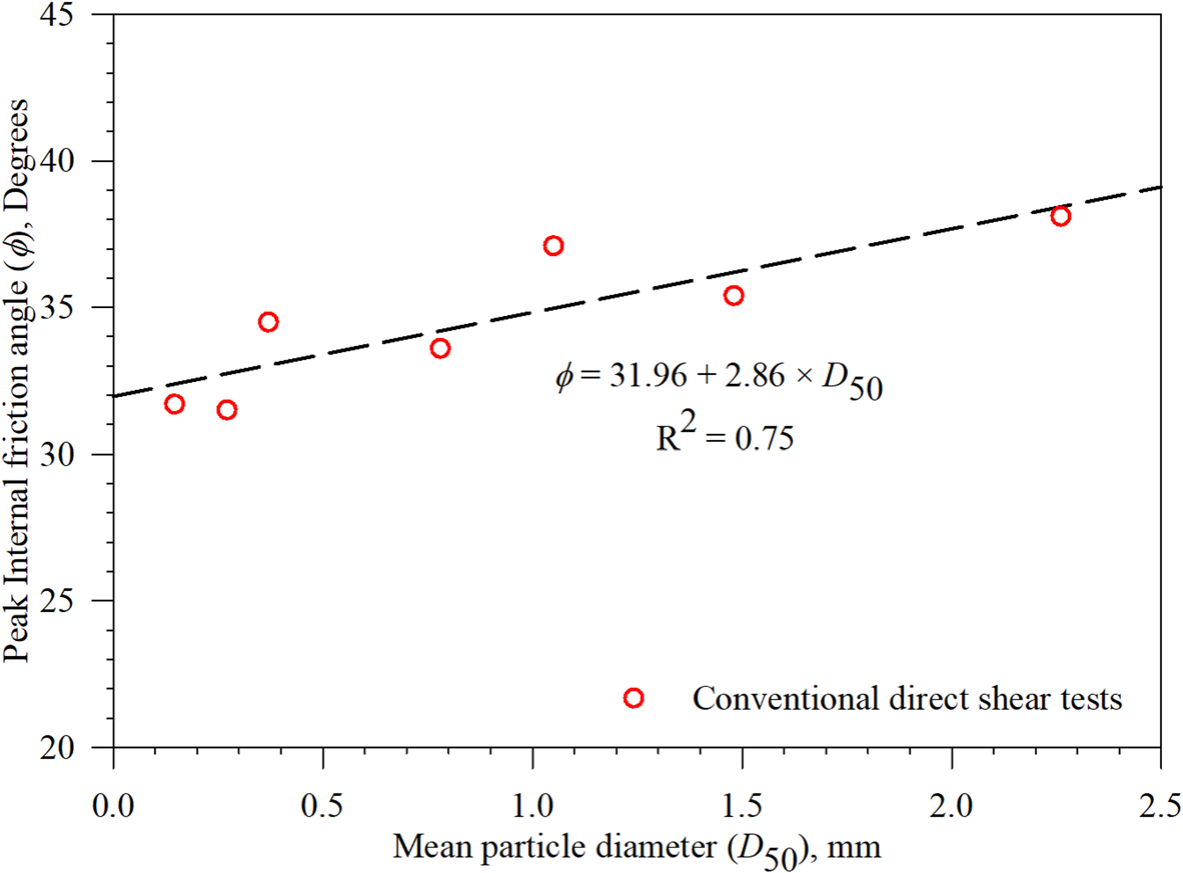
Effects of the partice size on the peak internal angle of friction of test sands (Sand 1 through Sand 7).

For all seven sands, three normal (confining) stresses of 100, 200, and 300 kPa were applied on the top of the sand specimens in conventional direct shear tests, while normal (confining) stress of 200 kPa is used in the interface direct shear tests. The measured shear stresses at peak were plotted against the corresponding normal (confining) stresses; the slope of the best fit line (i.e., linear regression line with zero intercept) for each group of tests was used to determine the peak internal friction angle (*ϕ*). Figure 7a-g presents the results of conventional direct shear tests on representative specimens of Sand 1 through Sand 7. Table 3 summarizes the values of peak friction angles (*ϕ*) for all test sands. Figure 8 shows the peak internal angle of friction (obtained from a series of conventional direct shear tests) of the silica sands, which have different particle sizes (as a function of the mean particle sizes *D*_50_). As shown in Fig. 8, the peak friction angle follows a linear relationship with mean particle diameter, the friction angle increases with increasing the mean particle size of the tested sand. The same phenomenon (relationship between friction angle and particle size) was observed by Han et al.^7^ from direct shear tests and drained triaxial tests performed on ten silica sands with mean particle diameters in the range between 0.23 mm and 2.68 mm.

**Table 3:** Mobilized interface shear stress.

Sand	Soil	Smooth		Textured 1		Textured 2		Textured 3	
	*ϕ* (°)	*τ* (kPa)	*τ*/*σ*	*τ* (kPa)	*τ*/*σ*	*τ* (kPa)	*τ*/*σ*	*τ* (kPa)	*τ*/*σ*
S1	31.7	80.0	0.400	120.0	0.600	140.0	0.700	130.6	0.650
S2	31.5	74.8	0.370	115.3	0.577	129.7	0.649	123.5	0.618
S3	34.5	80.0	0.400	117.3	0.587	127.6	0.638	133.7	0.668
S4	33.6	69.2	0.346	123.8	0.619	134.3	0.672	146.6	0.733
S5	37.1	81.9	0.409	127.9	0.639	153.8	0.769	172.7	0.864
S6	35.4	75.3	0.377	128.6	0.643	148.4	0.742	157.9	0.789
S7	38.1	75.6	0.378	143.4	0.717	165.3	0.827	178.2	0.891

### Effect of interface roughness

**Fig. 9:**
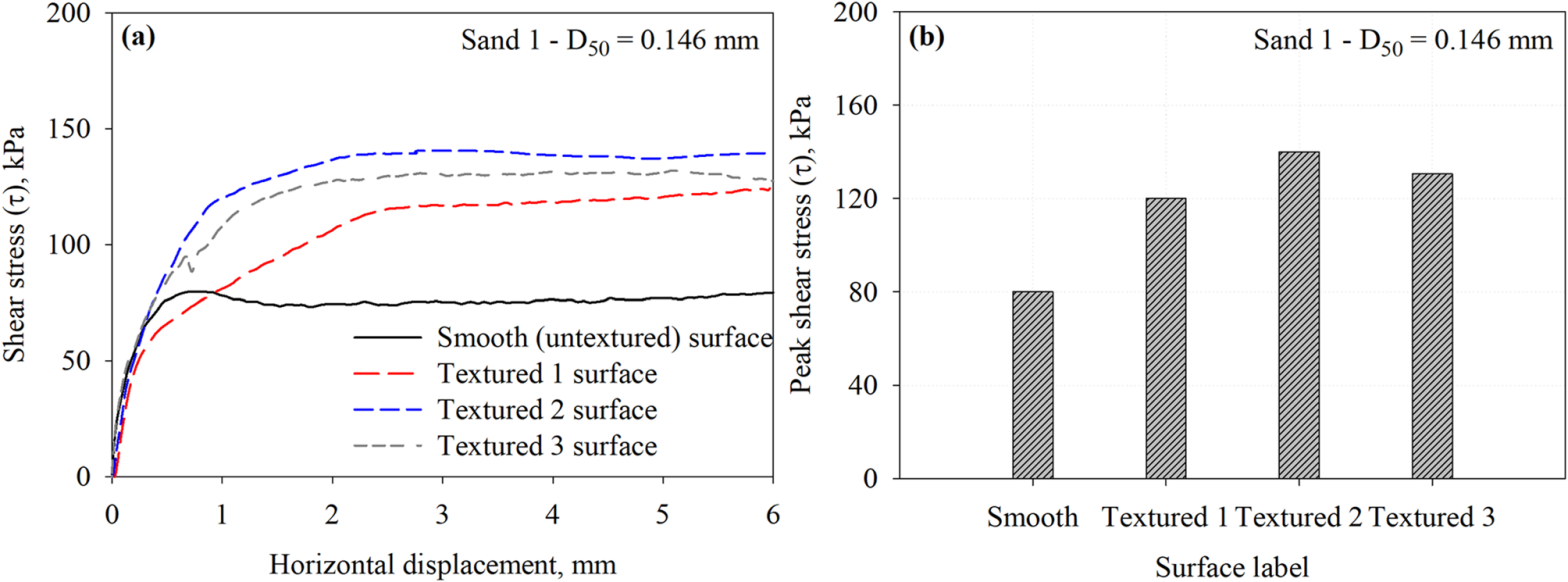
Results of interface tests performed on all surfaces in Sand 1 specimens under 200 kPa normal stress: (**a**) shear stress vs. horizontal displacement response curves and (**b**) maximum mobilized shear stress (*τ*) at the interface between Sand 1 and all surfaces.

Textured surface and a smooth surface were used to investigate the effect of surface element shape, spacing, and number on the interface shear stress. Figure 9 presents the results of a series of interface direct shear tests conducted on a smooth (untextured) surface and three textured surfaces with trapezoidal-like elements (see Table 2; Fig. 6) against representative specimens of Sand 1 under a normal stress of 200 kPa. All textured surfaces had an element height (*h*) 2.0 mm and element spacing (*S*) of 55 mm (Textured 1 with one element), 25 mm (Textured 2 with two elements), and 15 mm (Textured 3 with three elements), all tested in the direction perpendicular to the 90° side of the element (see Fig. 6). Figure 9a shows shear stress versus shear displacement curves, which clearly indicates that surface roughness significantly influences the interface shear strength. The Textured 2 surface mobilized the largest peak shear stress, followed by the Textured 3 surface, followed by the Textured 1 surface then by the smooth (untextured) surface. The low shear resistance observed for the smooth surface suggests that load transfer occurs primarily through friction (or sliding), with limited interlocking at the interface. Figure 9b presents the variation in peak shear stress mobilized at the interface between all surfaces and Sand 1 specimens. The measured values reveal a consistent increase in interface shear strength with increasing surface texturing up to a critical point, beyond which the strength decreases, highlighting the effectiveness of engineered surface roughness in enhancing soil–structure interaction. As suggested by Mitchell and Villet^32^ and Irsyam and Hryciw^31^, surfaces with elements or asperities protruding vertically could develop passive resistance in addition to friction, as the surface features force soil grains to displace under confining normal stress. Previous research studies reported that the contribution of passive resistance is influenced by the spacing between the elements or asperities. Other research studies^13,17,39,49,50^ suggested that asperity spacing to height (*S*/*h*) ratio controls the contribution of passive resistance to the total resistance, emphasizing that the maximum passive resistance occurs at optimum spacing or spacing to height ratio that allows the development of full passive soil wedges at the face of the transverse elements. The highest interface resistance for the Textured 2 surface (element to element spacing of 25 mm) maximizes the size and number of full passive wedges developed at the face of asperity per unit of contact, which maximizes the contribution of passive resistance to total resistance. Irsyam and Hryciw^31^ performed pull-out tests using a direct shear device on 2.5 mm square ribs in Ottawa 20–30 sand, it was found that the optimum spacing for loose and dense sand were 25 mm and 33 mm, respectively, thus solidifying the current findings. The interface shear resistance increases by 50.0, 75.0, and 63.3% for the Textured 1, Textured 2, and Textured 3 with respect to the smooth surface. The measured peak shear stresses at the interface with Sand 1 that has a *D*_50_ of 0.146 mm are summarized in Table 3.

**Fig. 10:**
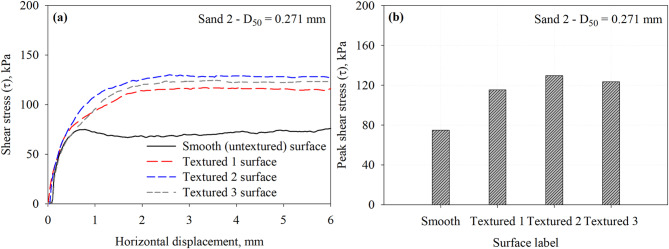
Results of interface tests performed on all surfaces in Sand 2 specimens under 200 kPa normal stress: (**a**) shear stress vs. horizontal displacement response curves and (**b**) maximum mobilized shear stress (*τ*) at the interface between Sand 2 and all surfaces.

Figure 10 presents the results of interface direct shear tests conducted on a smooth (untextured) and three textured surfaces against representative specimens of Sand 2 under a normal stress of 200 kPa. Figure 10a shows the shear stress versus shear displacement curves measured at the interface. The measured responses depict the influence of surface roughness on the interface shear strength, which is similar to the trends observed with Sand 1. Among the tested surfaces, the Textured 2 surface mobilized the highest peak shear stress, followed closely by the Textured 3 surface, then the Textured 1 surface, and lastly the smooth surface, which exhibited the lowest shear resistance.

Figure 10b presents the peak shear stress mobilized at the interface between each surface and Sand 2 (*D*_50_ = 0.146 mm). Similar to Sand 1, the highest interface resistance for the Textured 2 surface (element to element spacing of 25 mm) maximizes the size and number of full passive wedges developed at the face, which results in larger contribution of passive resistance to total resistance. The interface shear resistance increases by 54.1, 73.4, and 65.1% for the Textured 1, Textured 2, and Textured 3 with respect to the smooth surface. The measured peak shear stresses at the interface with Sand 2 that has a *D*_50_ of 0.271 mm are summarized in Table 3.

**Fig. 11:**
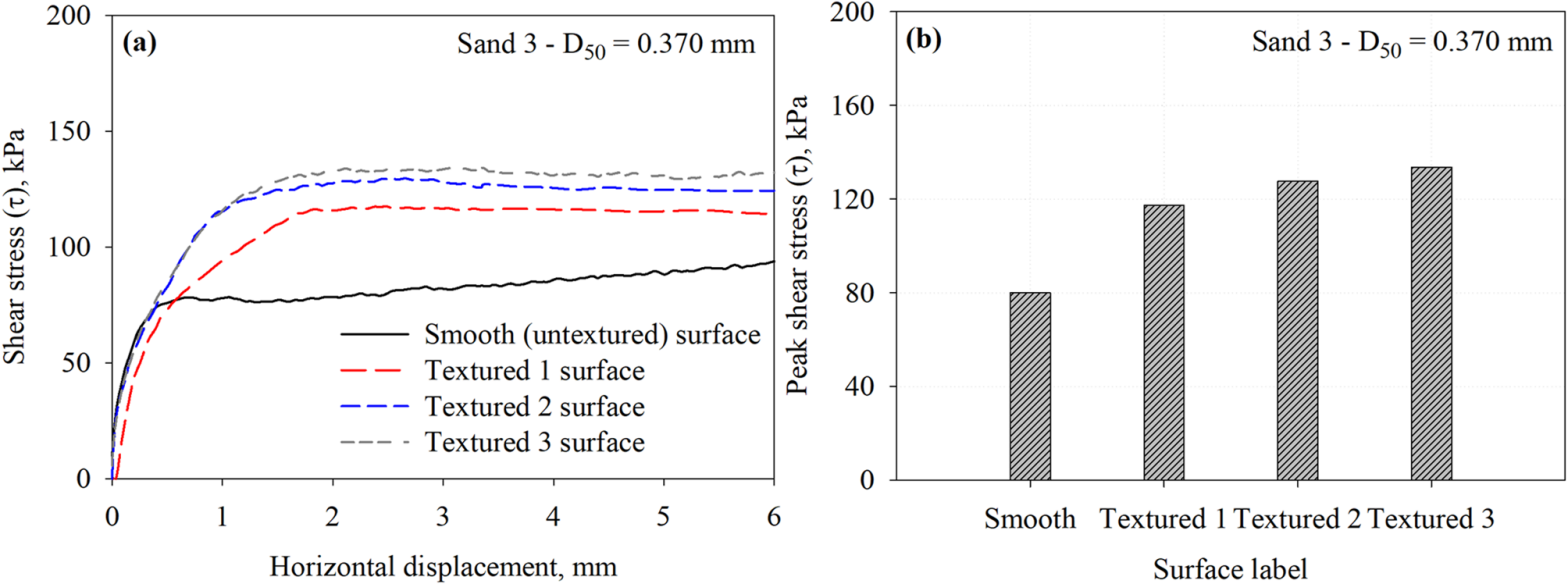
Results of interface tests performed on all surfaces in Sand 3 specimens under 200 kPa normal stress: (**a**) shear stress vs. horizontal displacement response curves and (**b**) maximum mobilized shear stress (*τ*) at the interface between Sand 3 and all surfaces.

Figure 11 presents the results of interface direct shear tests performed on the Smooth and the Textured 1, Textured 2, and Textured 3 against representative specimens of Sand 3 under a normal stress of 200 kPa. Figure 11a shows the shear stress versus horizontal displacement response curves at the interface. As shown in Fig. 11a, the Textured 3 surface mobilized the highest interface shear strength, followed by Textured 2, then Textured 1. Similar to Sand 1 and Sand 2, the Smooth surface mobilized the lowest interface shear resistance. Figure 11b shows that the mobilized peak shear resistance increases with number of elements, the Textured 3 surface achieved the highest interface shear resistance, indicating that the smaller element spacing of 15 mm, which corresponds to spacing to height (*S*/h) ratio of 7.5, might be sufficient for the development of full passive wedges at the face of surface elements. Compared to Sand 1 and Sand 2, this suggests that the optimum asperity spacing or asperity to height ratio for maximizing passive resistance may vary depending on the particle size and packing of the sand. The interface shear resistance increases by 46.6, 59.5, and 67.1% for the Textured 1, Textured 2, and Textured 3 with respect to the smooth surface. The measured peak shear stresses at the interface with Sand 3 that has a *D*_50_ of 0.370 mm are summarized in Table 3.

**Fig. 12:**
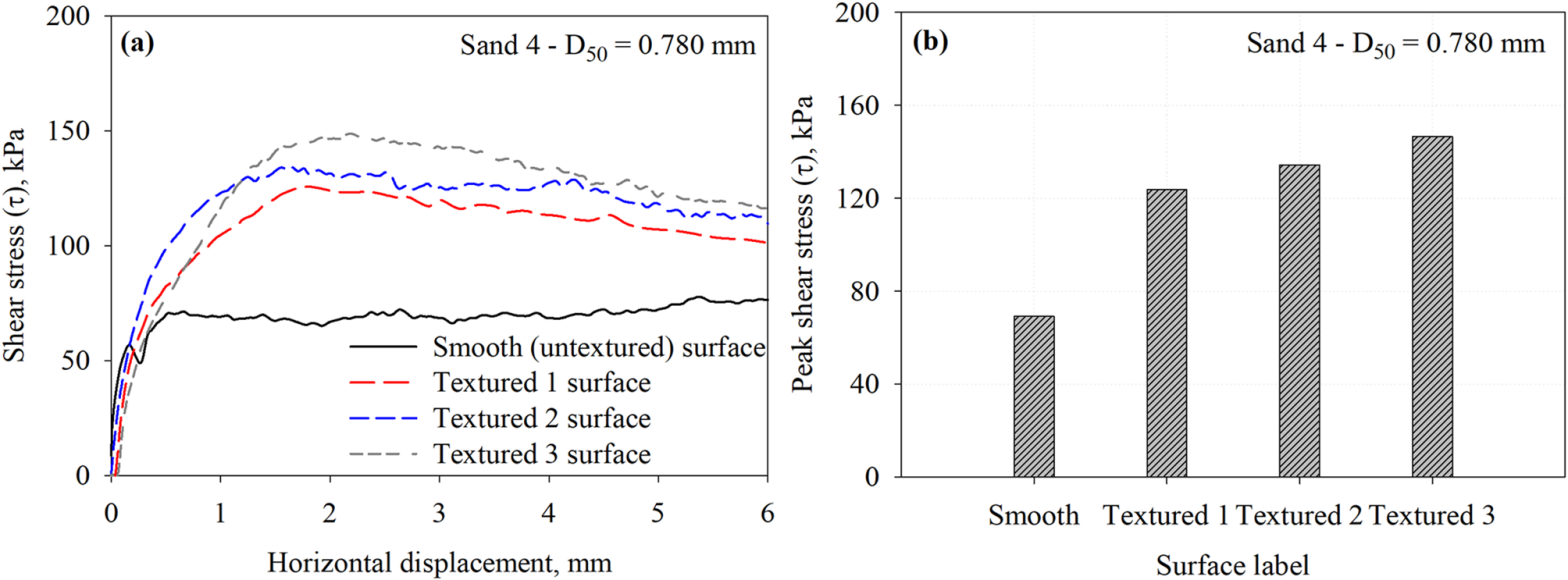
Results of interface tests performed on all surfaces in Sand 4 specimens under 200 kPa normal stress: (**a**) shear stress vs. horizontal displacement response curves and (**b**) maximum mobilized shear stress (*τ*) at the interface between Sand 4 and all surfaces.

Figure 12 presents the results of interface direct shear tests performed on a smooth (untextured) surface and three textured surfaces in contact with Sand 4 specimens under a normal stress of 200 kPa. The sand tested has a relatively coarse mean particle size (i.e., *D*_50_ = 0.780 mm). Figure 12a illustrates the shear stress versus horizontal displacement response curves for each surface, while Fig. 12b summarizes the peak shear stress values at the interface. As can be seen, all textured surfaces mobilized significantly higher peak shear stresses compared to the smooth surface, which highlights the role of surface characteristics (i.e., surface element height, shape and spacing) in the load transfer mechanisms (both frictional and passive resistance) at the interface.

An important observation from Fig. 12a is the clear post-peak softening behavior exhibited by all textured surfaces which is mostly pronounced in Textured 3. This softening indicates that after peak shear stress is reached, the resistance decreases with continued displacement. Such behavior suggests partial breakdown or rearrangement of the interlocking mechanisms (i.e., passive soil wedges) once the asperities are overcome or soil particles are displaced past the surface features. The degree of softening appears to be greater for the textured surface with more elements, reflecting a higher mobilization of passive resistance followed by its release. In contrast, the smooth surface shows a much flatter response, with very little softening consistent with a friction dominated interaction.

As observed from results conducted in Sand 4, the Textured 3 surface demonstrates the greatest interface strength, likely due to the high number of asperities (small spacing) allowing for multiple passive wedge formations per unit length. This suggests that for coarse-grained soils, smaller *S*/*h* ratios can increase passive resistance contribution to total resistance. It indicates that the grain size of test sand influences the optimum spacing required for the development of full passive zones at the face of asperity under shearing. The interface shear resistance increases by 78.9, 94.1, and 111.8% for the Textured 1, Textured 2, and Textured 3 with respect to the smooth surface. The measured peak shear stresses at the interface with Sand 4 that has a *D*_50_ of 0.780 mm are summarized in Table 3.

**Fig. 13:**
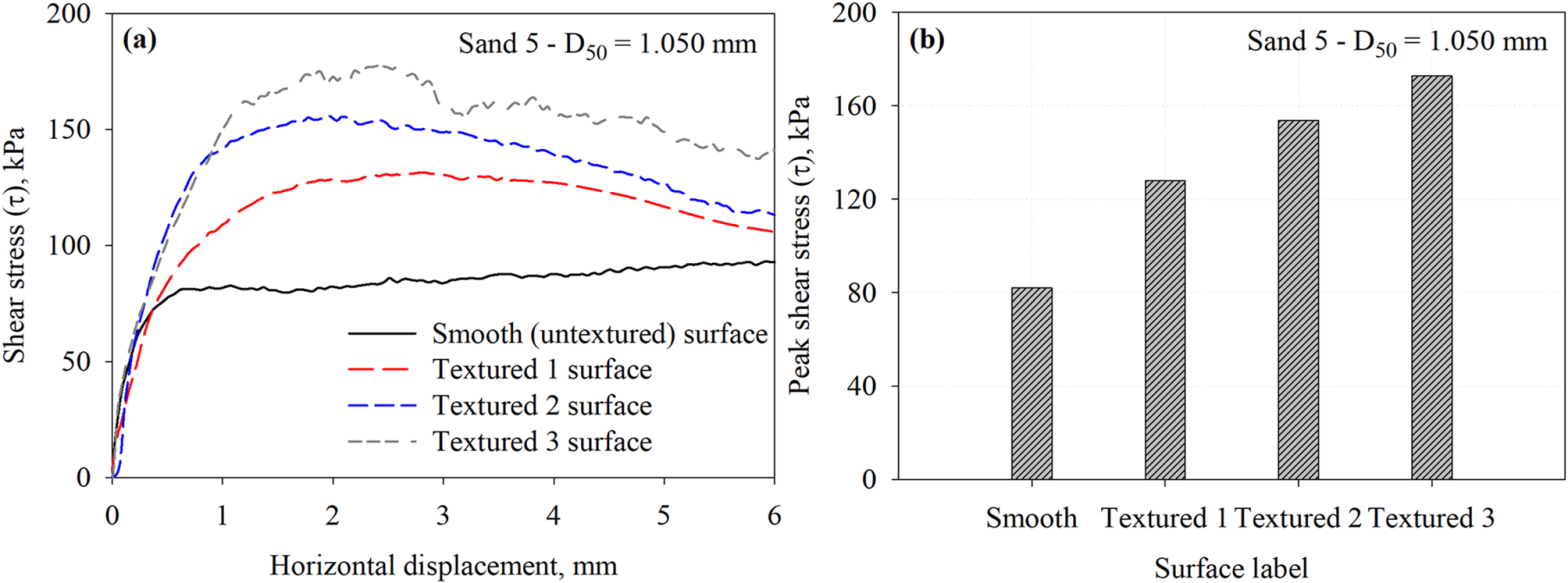
Results of interface tests performed on all surfaces in Sand 5 specimens under 200 kPa normal stress: (**a**) shear stress vs. horizontal displacement response curves and (**b**) maximum mobilized shear stress (*τ*) at the interface between Sand 5 and all surfaces.

Figure 13 shows the results of a series of interface shear tests on Sand 5 that has a mean particle diameter of 1.050 mm against the smooth and the Textured 1, Textured 2, and Textured 3 surfaces under a normal stress of 200 kPa. The Textured 3 exhibited a more pronounced post-peak softening behavior with shear displacement (Fig. 13a). Figure 13b summarizes the peak shear stress values at the interface. As illustrated in Fig. 13b, the interface shear resistance increases with number of elements and decreases with elements spacing. However, the asperity spacing to height ratio plays a crucial role in the interface shear resistance of textured surfaces in granular soils. The surface with *S*/*h* ratio of 7.5 mobilized that highest shear resistance among all surfaces. In other words, element to element spacing of 15 mm is sufficient to allow the development of passive wedges when compared to Textured 1 and Textured 2 surface that have spacing of 55 mm and 25 mm, respectively. The interface shear resistance increases by 56.2, 87.8, and 110.9% for the Textured 1, Textured 2, and Textured 3 with respect to the smooth surface. The measured peak shear stresses at the interface with Sand 5 that has a *D*_50_ of 1.050 mm are summarized in Table 3.

**Fig. 14:**
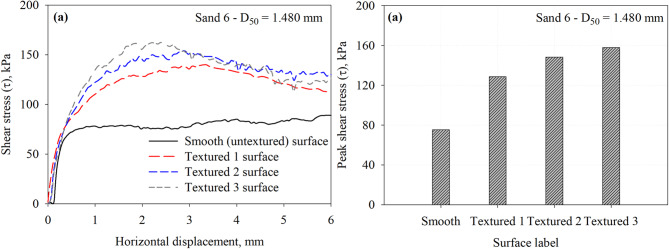
Results of interface tests performed on all surfaces in Sand 6 specimens under 200 kPa normal stress: (**a**) shear stress vs. horizontal displacement response curves and (**b**) maximum mobilized shear stress (*τ*) at the interface between Sand 6 and all surfaces.

Figure 14 reports the results of interface shear tests on smooth and three textured surfaces against the Sand 6 that has a mean particle diameter of 1.480 mm. The tests were performed under a normal stress of 200 kPa. Unlike the smooth surface, the textured surface post-peak softening behavior with shear displacement (Fig. 14a). Similar behavior was observed by Han et al.^7^ from tests on silica sand with a mean particle diameter of 2.68 mm tested against random rough surface.

Figure 14b shows the values of peak shear stress mobilized at the interface. As illustrated in Fig. 14b, the interface shear resistance increased with level of roughness (i.e., number of elements per unit of contact length), which results in smaller element to element spacing. The Textured 3 surface (asperity height of 2.0 mm, spacing of 15 mm, *S*/*h* ratio of 7.5) mobilized the highest resistance when compared to smooth, Textured 1, and Textured 2 surfaces. The element to element spacing of 15 mm (corresponds to *S*/*h* ratio of 7.5) allows the development of full passive wedges or maximize the size of soil wedges when compared to Textured 1 and Textured 2 surface that have spacing of 55 mm and 25 mm, respectively. The Textured 2 surface mobilized interface shear resistance that is larger than the one mobilized by Textured 1 surface. The element to element spacing in Textured 2 surface allows for larger passive resistance contribution to the total resistance. The smooth (untextured) surface mobilized low resistance through friction (or sliding) with shear displacement. The interface shear resistance increases by 70.8, 97.1, and 109.7% for the Textured 1, Textured 2, and Textured 3 with respect to the smooth surface. The measured peak shear stresses at the interface with Sand 6 that has a *D*_50_ of 1.480 mm are summarized in Table 3.

**Fig. 15:**
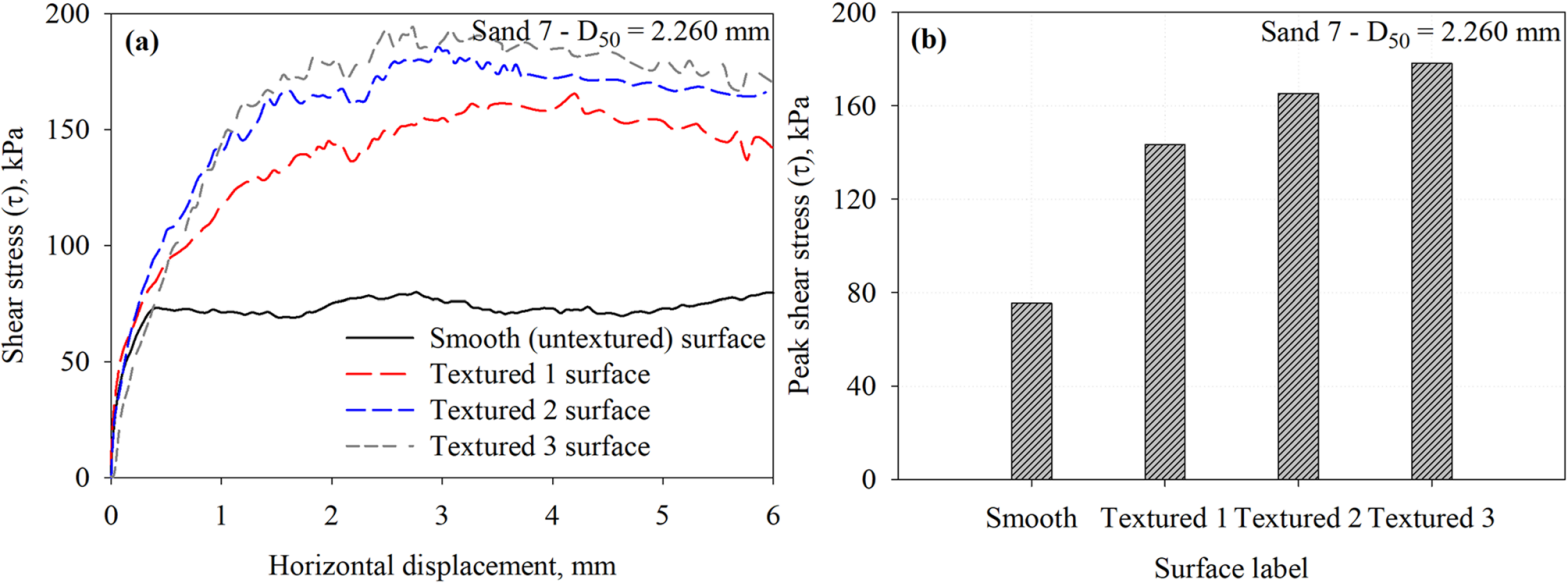
Results of interface tests performed on all surfaces in Sand 7 specimens under 200 kPa normal stress: (**a**) shear stress vs. horizontal displacement response curves and (**b**) maximum mobilized shear stress (*τ*) at the interface between Sand 7 and all surfaces.

Results from interface shear tests conducted on representative specimens of Sand 7, which has a mean particle diameter of 2.260, against the smooth and three textured surfaces under a normal stress of 200 kPa are shown in Fig. 15. As shown in Fig. 15a, the rough surfaces exhibited post-peak softening behavior with shear displacement (Fig. 15a). Similar behavior was observed by Han et al.^30^ from tests on silica sand with a mean particle diameter of 2.68 mm tested against random rough surface. As shown in Fig. 15b, Textured 3 surface mobilized the highest interface shear resistance, followed by Textured 2, then Textured 1. Textured 3 surface has element to element spacing of 15 mm, the spacing allows the development of passive soil wedges at the face of elements which result in larger interface shear resistance. Similarly, smooth (untextured) surface mobilized low resistance through friction (or sliding) with shear displacement. The interface shear resistance increases by 89.7, 118.7, and 135.7% for the Textured 1, Textured 2, and Textured 3 with respect to the smooth surface. The measured peak shear stresses at the interface with Sand 7 that has a *D*_50_ of 2.260 mm are summarized in Table 3. It is important to note that negligible crushing of test sands or scratching of the test surfaces was observed during interface shear testing. Xiao et al.^25^ who conducted a series of interface shear tests between calcareous sand and steel surfaces with asymmetric, morphologic profiles inspired by ventral snake scales. Calcareous sand has angular particle shapes and abundant inner pores, which make it more prone to particle crushing^25^. They concluded that the particle breakage of the calcareous sand under interface shearing is affected by the normal stresses developed and the geometry of the steel surface. A normal stress level up to 800 kPa was used to evaluate the evolution of particle breakage under interface shearing. Xiao et al.^25^ compared the original gradation curves of test sand to after interface tests curves at normal stresses up to 800 kPa. For example, Han et al.^7^ studied the effects of interface roughness and particle size on the interface friction angle for rusted steel, the interface was subjected to normal stresses of 100, 200, and 300 kPa. Stutz and Martinez^24^ investigated the interface shear behavior of surfaces with asymmetric profiles inspired by the scales of snakeskin, the interface tests were conducted under normal stresses of 75, 125, and 200 kPa, respectively. Several research studies^54–57^ have examined the influence of particle breakage during interface shearing on gravelly soil-structure interfaces, showing that grain fracture markedly alters both the mobilized shear resistance and the deformation response of these interfaces. Previous studies on silica sand tested against surfaces with random and structured roughness form under comparable normal stress levels reported that particle breakage is negligible^7,24^.

### Effect of particle size

**Fig. 16:**
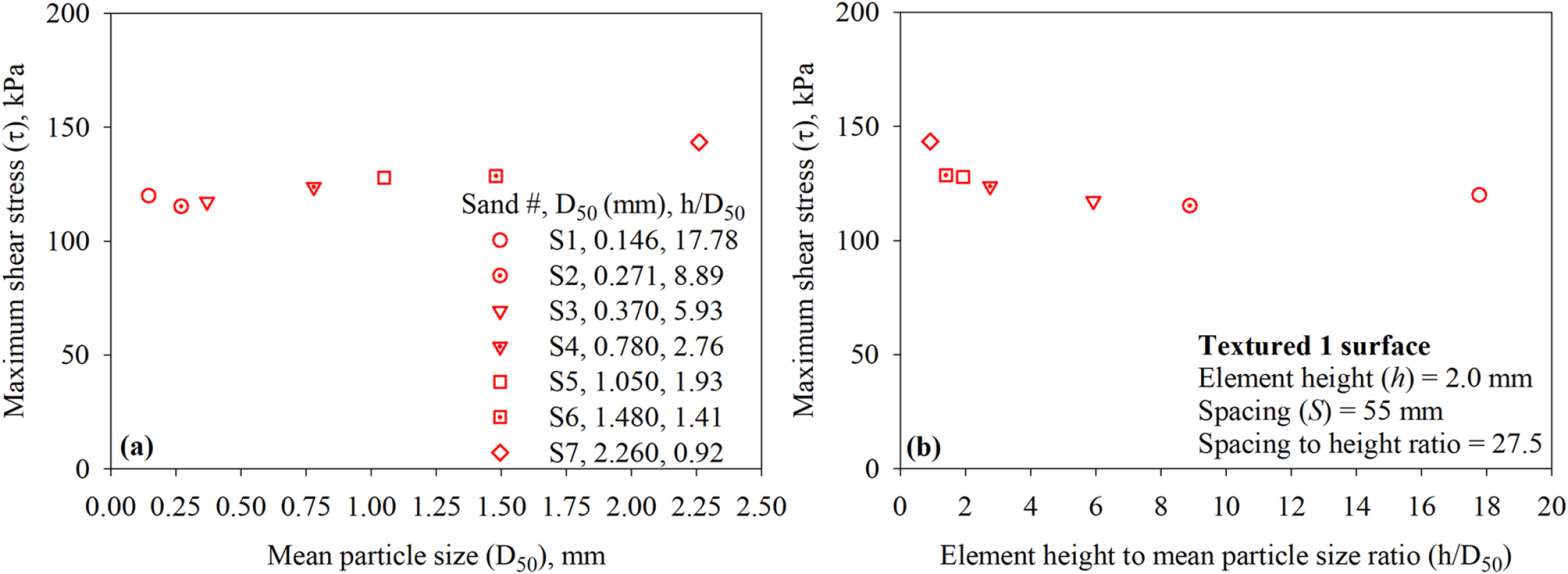
Effect of sand particle size on the mobilized interface shear stress for textured 1 surface under 200 kPa normal stress: (**a**) maximum shear stress (*τ*) versus mean particle size (*D*_50_); and (**b**) maximum shear stress (*τ*) versus element height to mean particle size ratio (*h*/*D*_50_) for all sands.

For a given surface (with structured asperities or elements) tested against sandy soils, the maximum interface shear stress (*τ*) is a function of the sand particle size. Figure 16a presents the maximum interface shear stress (*τ*) for the Textured 1 surface (with one trapezoidal-like element) versus the mean particle size (*D*_50_) of all test sands (sand 1 through 7). As shown in Fig. 16a, the maximum mobilized shear resistance at the interface between sand and Textured 1 surface is found to be increasing with the increase in the mean particle diameter (*D*_50_). The results reported herein contradict the findings of previous research studies on the shear behavior of surfaces with random roughness in cohesionless (sandy) soils^7^. Han et al.^60^ who performed interface direct shear tests on smooth steel, lightly rusted steel, rusted steel, and heavily rusted steel in sandy soil indicated that the interface shear resistance (interface friction angle) decreases as the particle size increases. The interface shear resistance (interface friction angle) increases with increasing the normalized roughness (*R*_*n*_) of a given surface until it exceeds a threshold value. For a given surface with constant maximum roughness, *R*_*n*_ increases as the particle size decreases^2^. A normalized roughness parameter, defined as the ratio of element or asperity height to mean particle diameter (*R*_*n, structued*_ = *h/D*_50_), is introduced herein in an effort to capture the coupling effect of the roughness of a structured surface and the particle size of sand on the interface shear resistance under monotonic loading. The *h/D*_50_ ratio appears to unify the data from the tests against the textured or structured surfaces. The experimental results of the tests on Textured 1 surface against the seven sands (shown in Fig. 16b) suggest that the shear resistance mobilized at the interface decreases with increasing normalized structured roughness (*R*_*n, structued*_ = *h/D*_50_ or element height to mean particle diameter ratio) or decreasing *D*_50_ for the same asperity height. A comparison of trends obtained with the *h/D*_50_ in the present study and *R*_*n*_ in previous studies^2,7,50,58^ suggests that the *h/D*_50_ ratio is better suited to capture the load-transfer mechanism at the interface between sand and surfaces with structured elements such as the development of passive resistance in addition to friction resistance.

As suggested by other researchers^5,7,12,50,59^, roughness parameters such as average surface roughness (*R*_*a*_) or normalized roughness (*R*_*n*_) are well-suited to describe the relationship between the shear strength at interface between randomly rough surfaces and sandy soils. Such surfaces are commonly produced from abrasion or rust. As shown in Fig. 16b, the maximum interface resistance of 143.4 kPa (*τ/σ* = 0.717 and *δ/φ* = 0.935) mobilizes at a *h/D*_50_ ratio of 0.92, the *h/D*_*50*_ ratio of 0.92 corresponds to asperity height of 2.0 mm and *D*_50_ of 2.260 mm (Sand 7 or coarse sand). According to Uesugi and Kishida^30^, it is anticipated for a random surface with constant roughness (in scale of µm) tested against coarse sand with large *D*_50_ to mobilize low shear strength (i.e., low normalized roughness). However, the opposite is observed for surfaces with structured elements with constant roughness (in scale of mm) when tested against coarse sand.

**Fig. 17:**
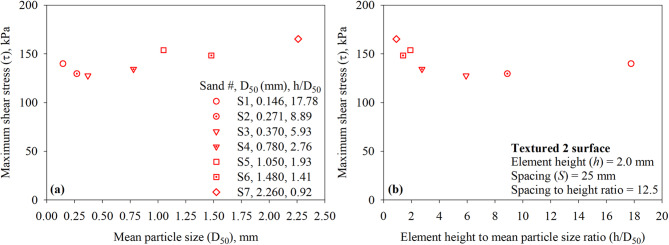
Effect of sand particle size on the mobilized interface shear stress for textured 2 surface under 200 kPa normal stress: (**a**) maximum shear stress (*τ*) versus mean particle size (*D*_50_); and (**b**) maximum shear stress (*τ*) versus element height to mean particle size ratio (*h*/*D*_50_) for all sands.

Figure 17 shows the results of interface direct shear tests performed on Textured 2 surface against all seven sands (sand 1 through 7). Like Textured 1 surface, the maximum shear stress at the interface of Textured 1 surface (with two trapezoidal-like elements) found to be increasing with the increase in the mean particle diameter (*D*_50_). The data reported in Fig. 17a contradicts the findings of previous research studies on the shear behavior of surfaces with random roughness in cohesionless (sandy) soils^7^. As shown in Fig. 17b, the *h/D*_50_ ratio appears to unify the data from the tests against the textured or structured surfaces. The data plotted in Fig. 17b suggest that the shear resistance mobilized at the interface decreases with increasing normalized structured roughness (*R*_*n, structued*_ = *h/D*_50_ or element height to mean particle diameter ratio) or decreasing *D*_50_ for the same asperity height. The trend observed indicates that the *h/D*_50_ might be a well-suited controlling factor over the relationship between the shear strength at interface between random surfaces and sandy soils. A comparison of these trends (from tests on Textured 1 and Textured 2 surfaces) indicate that the *h/D*_50_ controls the total interface shear resistance in cohesionless (sandy) soils. As shown in Fig. 17b, the maximum interface resistance of 165.3 kPa (*τ/σ* = 0.827 and *δ/ϕ* = 1.038) mobilizes at a *h/D*_50_ ratio of 0.92, the *h/D*_50_ ratio of 0.92 corresponds to asperity height of 2.0 mm and *D*_50_ of 2.260 mm (Sand 7 or coarse sand).

**Fig. 18:**
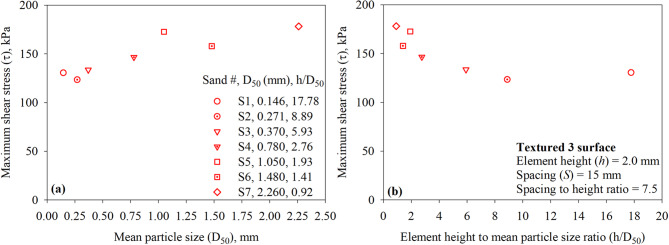
Effect of sand particle size on the mobilized interface shear stress for textured 3 surface under 200 kPa normal stress: (**a**) maximum shear stress (*τ*) versus mean particle size (*D*_50_); and (**b**) maximum shear stress (τ) versus element height to mean particle size ratio (*h*/*D*_50_) for all sands.

Figure 18 presents the results of interface direct shear tests on Textured 3 surface against all seven test sands (sand 1 through 7). As observed from tests on Textured 1 and Textured 2 (see Figs. 16 and 17), the maximum shear stress at the interface of Textured 3 surface (with three trapezoidal-like elements) found to be increasing with the increase in the mean particle diameter (*D*_50_). Results plotted in Fig. 18b indicate that the interface shear resistance of surfaces with more trapezoidal-like elements decreases with increasing normalized structured roughness (*R*_*n, structued*_ = *h/D*_50_ or element height to mean particle diameter ratio) or decreasing *D*_50_ for the same asperity height. A comparison of the trends between maximum shear stress (*τ*) and *h/D*_50_ among all surfaces suggests that the load-transfer mechanism at the interface between surfaces with structured roughness and cohesionless (sandy) soil is influenced by the newly introduced normalized structured roughness (*R*_*n, structued*_ = *h/D*_50_).

**Fig. 19:**
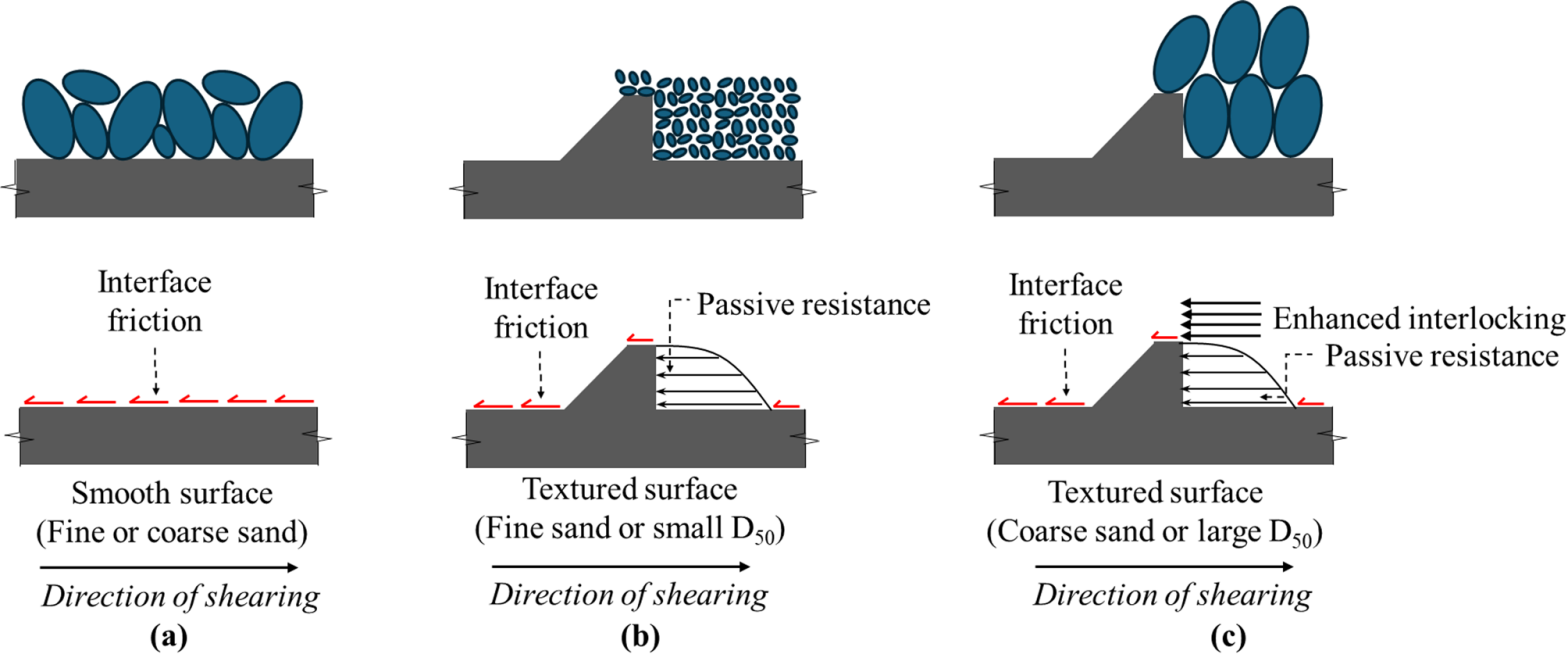
Simple interpretation of load transfer at the interface: (**a**) sand-smooth surface; (**b**) fine sand-textured surface; and (**c**) coarse sand-textured surface.

The surfaces proposed in the present study are surfaces with structured millimeter-scale roughness. Based on the conclusion by Han et al.^60^ that sand with mean diameter close to the asperity peak-to-valley distance (random micrometer-scale roughness) exhibits higher interface friction angles. Therefore, it is expected for the sand with the *D*_50_ of 2.260 mm (i.e., coarse or S7) close to the asperity or element height (h) of 2.0 to exhibit higher interface friction angles as it leads to enhanced interlocking at the interface. For surface with same asperity or element height, it is expected for the mobilized shear resistance to increase with particle mean diameter. The data reported herein also contradicts the findings of research studies on random rough surfaces^7^. The reason for this different behavior can be understood by referring to Fig. 19, where the graphical interpretation of roughness is shown. For sand-smooth surface (Fig. 19a), the load-transfer occurs mainly through sand to surface sliding^2,30,40^. For the fine sand or small *D*_50_ (Fig. 19b), the asperity height to sand diameter ratio enables friction and passive resistance. For the coarser sand or large *D*_50_ (Fig. 19c), the asperity height to sand diameter ratio enables friction and passive resistance in addition to enhanced interlocking at the interface during shearing. The experimental results of the present study suggest that the interface shear resistance of structed surfaces maximize when the *h*/*D*_50_ approximately 1(i.e., the mean particle size (*D*_50_) of the sand fairly matches with the asperity size). The observed behavior is consistent with the findings reported by Vangla and Latha Gali^60^. Vangla and Latha Gali^60^ investigated the effect of particle size of sand on the surface asperities of reinforcing material on the interface shear strength through conducting interface direct shear tests on sand-geotextile and sand wire-mesh against fine, medium, and coarse sands. They concluded that higher interfacial shear strength can be obtained when the mean particle size (*D*_50_) of the sand fairly matches with the asperity size of the interfacing material because of better interlocking achieved by the sand particles closely fitting into the asperities^60^.

### Effect of element characteristics

**Fig. 20:**
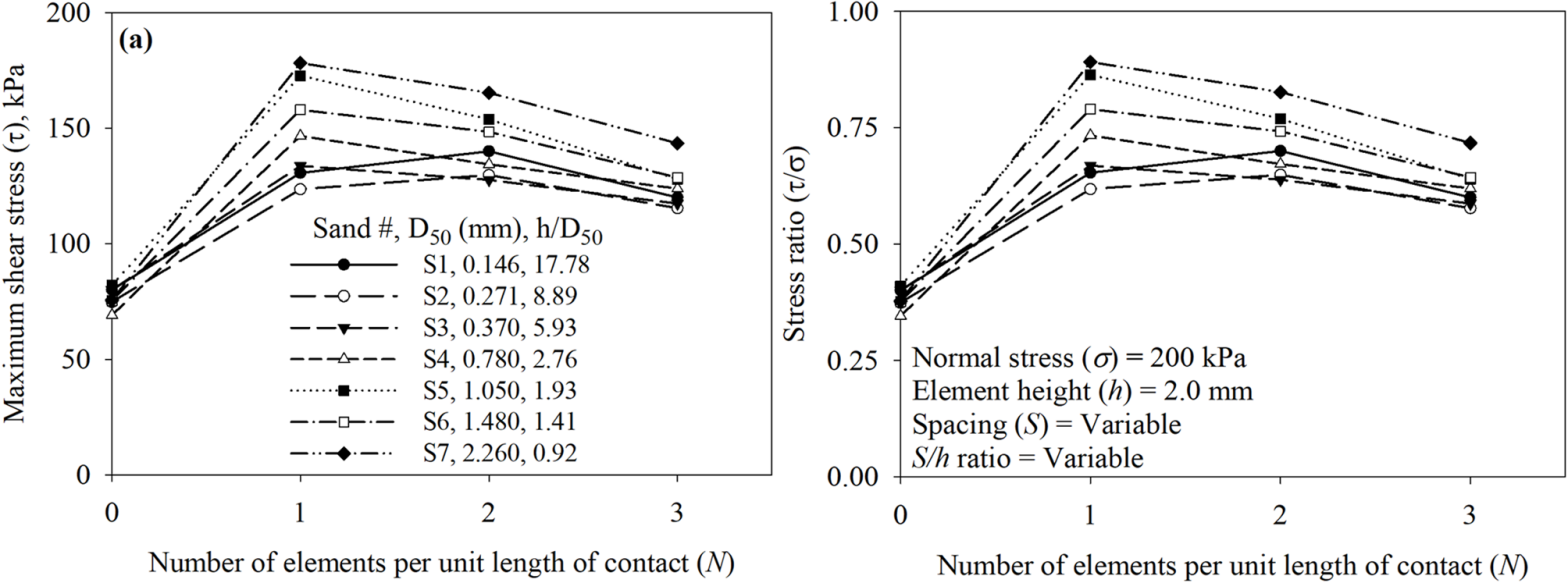
Effect of surface design on the mobilized interface shear stress of all surfaces under 200 kPa normal stress: (**a**) maximum shear stress (*τ*) versus number of elements (*N*), and (**b**) maximum shear stress ratio (*τ/σ*) versus number of elements (*N*).

A parametric study on the effect of the number of elements per unit length of contact (*N*), element-to-element asperity height (*S*), and asperity spacing to height (*S/h*) was executed to investigate the independent effect of these parameters on the shear behavior and response. Increases in *N* (as *N* increases *S* decreases) while keeping *h* constant at 2.0 mm resulted in an increase in the maximum mobilized shear resistance as shown in Fig. 20a and b for tests on all seven sands (Sand 1 through 7). All surfaces had asperity height (*h*) of 2.0 mm and were tested in the direction of the right-angle side of the trapezoidal-like elements (See Fig. 6). Tests against Sand 1 (*D*_50_ = 0.146 mm) and Sand 2 (*D*_50_ = 0.271 mm) indicated that the surface with two elements (labeled as Textured 2) mobilized the highest shear resistance, whereas tests against sands (Sand 3 through 7) (See Table 1 for *D*_50_ values) indicated that the surface with a one element (labeled as Textured 1) mobilized the highest shear resistance among all surfaces. The relationship between the maximum shear stress (*τ*) and stress ratio (*τ/σ*) and the number of surface elements (*N*) per unit of contact length is shown in Fig. 20. As shown in Fig. 20a and b, the mean particle diameter (*D*_50_) of the test sand has a significant effect on the maximum shear stress (*τ*) and stress ratio (*τ/σ*) mobilized at the interface with textured surfaces. When the number of elements per unit length of contact (*N*) is 1, representing largely spaced elements, the shear response varies markedly with sand type (size). Finer sands (e.g., S1 and S2) mobilize lower shear stress compared to coarser sands (e.g., S6 and S7), particularly at lower values of *N*. As *N* increases, the differences in mobilized shear stress among the sands diminish, suggesting that closely spaced elements reduce the dependency of shear strength on particle size. This trend may be attributed to the reduced spacing behind the elements, which limits the formation of passive soil wedges, thereby leading to additional and more uniform passive resistance across the test sands.

**Fig. 21:**
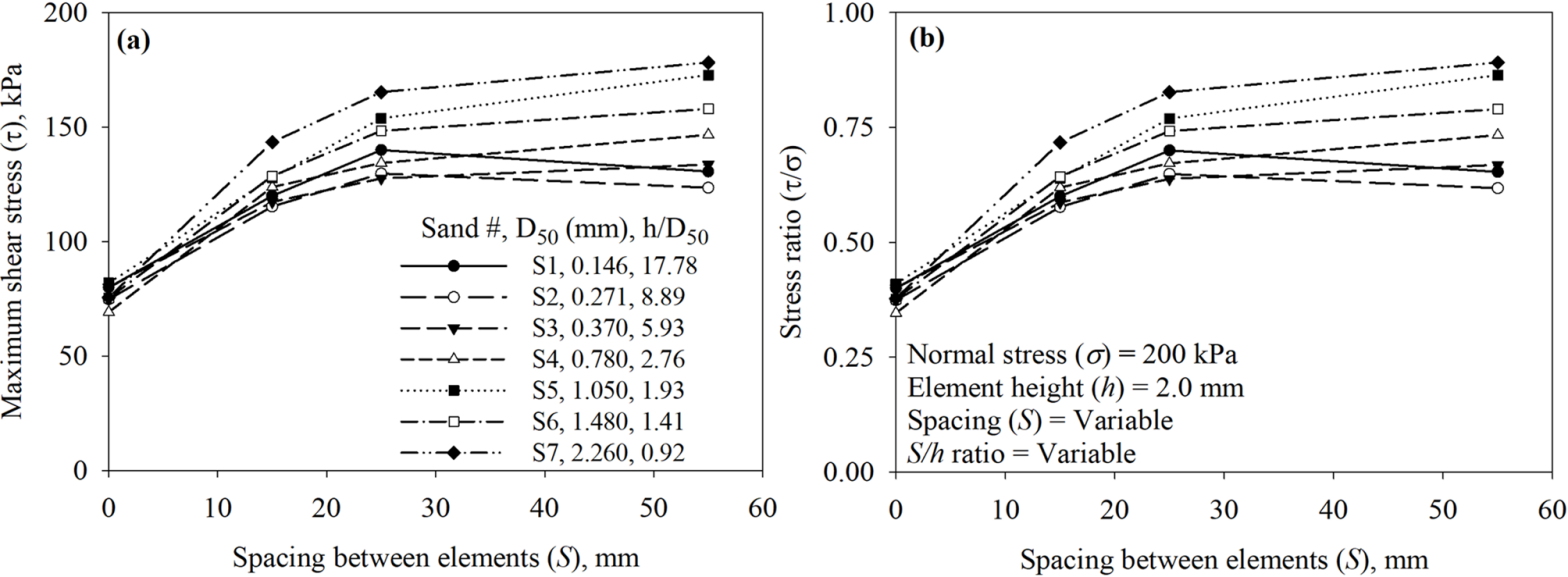
Effect of surface design on the mobilized interface shear stress of all surfaces under 200 kPa normal stress: (**a**) maximum shear stress (*τ*) versus spacing between elements (*S*), and (**b**) maximum shear stress ratio (*τ/σ*) versus spacing between elements (*S*).

Increases in *S* (as *S* increases *N* decreases) while keeping *h* constant at 2.0 mm resulted in an increase in the maximum shear resistance as shown in Fig. 21a and b. All surfaces had asperity height (*h*) of 2.0 mm and were tested in the direction of the right-angle side of the trapezoidal-like element (See Fig. 6). Tests against Sand 1 (*D*_50_ = 0.146 mm) and Sand 2 (*D*_50_ = 0.271 mm) indicated that the surface with *S* of 25 mm (labeled as Textured 2) mobilized the highest shear resistance, whereas tests against sands (Sand 3 through 7) (See Table 1 for *D*_50_ values) indicated that the surface with *S* of 55 mm (labeled as Textured 1) mobilized the highest shear resistance among all surfaces. The relationship between the maximum shear stress (*τ*) and stress ratio (*τ*/*σ*) and the element-to-element spacing (*S*) is shown in Fig. 21. As shown in Fig. 21a and b, the mean particle diameter (*D*_50_) of the test sand has a significant effect on the maximum shear stress (*τ*) and stress ratio (*τ/σ*) mobilized at the interface with textured surfaces. When the spacing between elements per unit length of contact (*S*) is 55 mm, representing largely spaced elements, the shear response varies markedly with sand type (size). Finer sands (e.g., S1 and S2) mobilize lower shear stress compared to coarser sands (e.g., S6 and S7), particularly at smaller values of *S*. As *S* decreases, the differences in mobilized shear stress among the sands increase, suggesting that closely spaced elements reduce the dependency of shear strength on particle size. This trend may be due to the reduced spacing behind the elements, which limits the formation of passive soil wedges, thereby resulting in more uniform additional passive resistance for test sands.

**Fig. 22:**
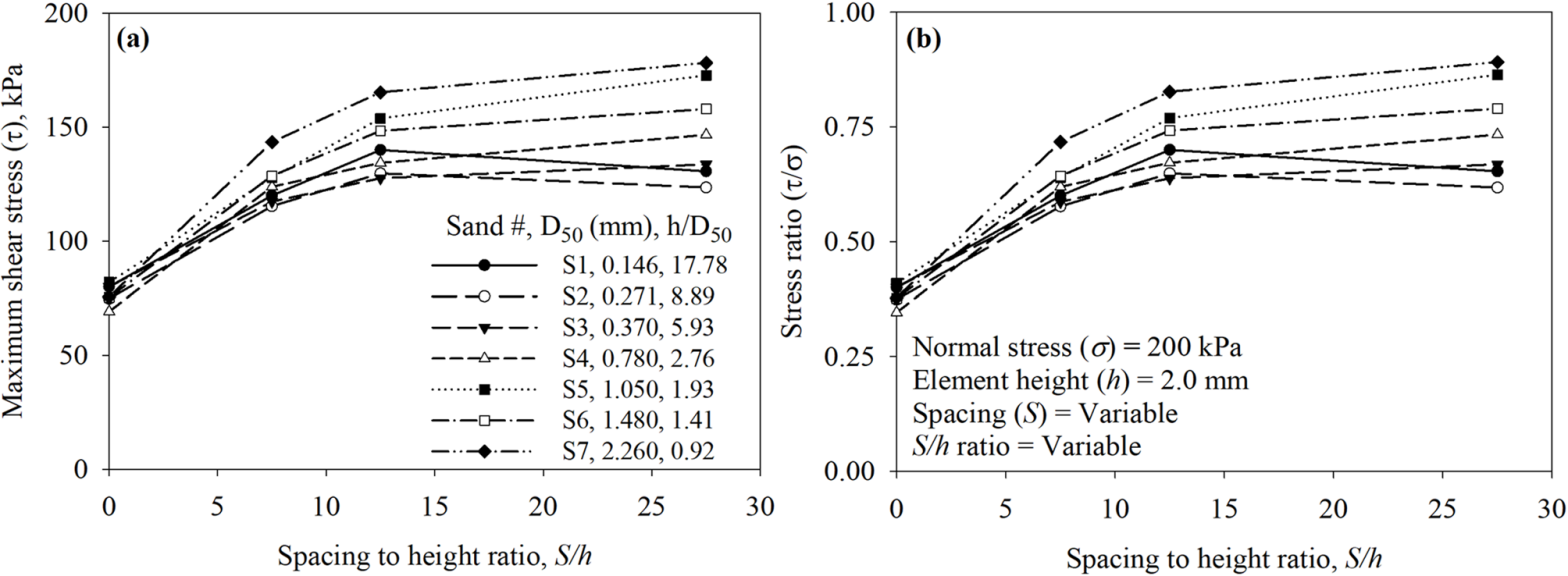
Effect of surface design on the mobilized interface shear stress of all surfaces under 200 kPa normal stress: (**a**) maximum shear stress (*τ*) versus spacing to height ratio (*S*/*h*), and (**b**) maximum shear stress ratio (*τ*/*σ*) versus spacing to height ratio (*S*/*h*).

The results of all the interface direct shear tests against smooth and textured surfaces are plotted as a function of *S/h* in Fig. 22a and b for all seven sands (Sand 1 through 7). The *S/h* ratio appears to unify the data from the tests against the surfaces, the test results highlight the impact of particle size on the mobilized shear resistance at the interface. For fine sands (Sand 1 and Sand 2), a surface with structured elements that has *S/h* ratio of 12.5 attains the highest shear resistance, whereas for medium to coarse sands (Sand 3 through 7), a surface with structured elements that has *S/h* ratio of 27.5 attains the highest shear resistance. The highest shear resistance of surface against fine sands implies that smaller spacing (*S* of 25 mm between two elements) is sufficient to develop full passive wedges at the face of the elements, while coarser sands require larger spacing (*S*) of 55 mm for the development of passive wedges during monotonic shearing. Surface with smaller spacing of 15 mm between surface elements (e.g., Textured 3) allows the development of partial passive wedges which minimize the contribution of passive resistance to total resistance when compared to Textured 1 and Textured 2 surfaces. As shown in Fig. 22a and b, the mean particle diameter (*D*_50_) of the test sand has a significant effect on the maximum shear stress (*τ*) and stress ratio (*τ/σ*) mobilized at the interface with textured surfaces. When the spacing to height ratio (*S/h*) is 27.5 mm, representing largely spaced elements, the shear response varies markedly with sand type (size). Finer sands (e.g., S1 and S2) mobilize lower shear stress compared to coarser sands (e.g., S6 and S7), particularly at smaller values of *S/h*. As *S/h* increases, the differences in mobilized shear stress among the sands increase, suggesting that widely spaced elements increase the dependency of shear strength on particle size. The small *S/h* values represent closely spaced elements (i.e., limit space for soil wedges developed at the face of elements), which results in more uniform additional passive resistance for test sands.

### Practical implications

The results presented herein indicate that tests with textured surfaces and a smooth (untextured) surface performed against seven poorly-graded sands of different particle sizes mobilized shear resistances consist of friction and passive resistance of different magnitudes. The element-to-element spacing (*S*) and spacing to element height ratio (*S/h*) of the textured surfaces with trapezoidal-like elements also influenced the mobilized shear resistances. Unlike shear resistances of surfaces with random roughness that is controlled by the normalized roughness parameter (*R*_*n*_) introduced by Uesugi and Kishida^2,30^, the shear resistance of structured surfaces is found to be controlled by the mean particle diameter (*D*_50_), newly introduced element height to mean particle diameter parameter *h*/*D*_50_, element-to-element spacing (*S*) and spacing to element height ratio (*S/h*) under monotonic shearing in sandy soils. A comparison of trends obtained with element height ratio (*S/h*) and element height to mean particle diameter parameter *h*/*D*_50_ suggest that these parameters could better capture interface shear-transfer mechanisms induced by surfaces with structured roughness elements. The results presented herein contradicts the widely accepted relationship between roughness parameters such as *R*_*a*_, *R*_max_, and *R*_*n*_ and interface strength mobilized for surfaces with random form produced by processes such as abrasion and rust^2,7^. Future research is required to address the extended role of considered parameters on the cyclic shear strength and frictional anisotropy during monotonic and cyclic shearing on coarse-grained and fine-grained soils.

## Conclusions

Conventional direct and interface direct shear tests were carried out with seven silica sands on smooth polished (untextured) interface, and textured interfaces with trapezoidal-like elements of same height but different numbers. The characteristics of all test sands and the surface elements characteristics (form, spacing, and height) were used to study their coupling effects on the interface shear response. The results highlight the following trends in maximum shear stress (*τ*) and stress ratio (*τ*/*σ*).

- The structured roughness plays an important role in the interface shear strength, the textured surfaces mobilized shear resistance up to 2.36 times the resistance mobilized by the smooth (untextured) surface, which was mobilized at the interface Textured 3 surface and Sand 7 (coarser sand). The shape of the element influenced the magnitude of the mobilized resistance when shearing the right-angle side of the trapezoidal-like element against sand allowed the development of additional passive resistance. The contribution of the passive resistance to total resistance appeared to be influenced by the spacing between elements (*S*) as well as the spacing to height (*S*/*h*) ratio.
- A newly introduced parameter, the normalized structured roughness (*R*_*n, structued*_ = *h/D*_50_), was shown to successfully unify the mobilized resistance and qualitative capture the shear load-transfer mechanism at the interface between textured surfaces with structured roughness and granular soils. For a textured surface with the same height element and spacing, small *h/D*_50_ values describe surfaces more likely induce large shear resistance. On the other hand, large *h/D*_50_ values correspond to surfaces that are more likely to mobilize smaller shear resistance.
- The element-to-element spacing (*S*) which varies with number of elements per unit of contact length (*N*), and element height (*h*) of the surfaces with structured roughness significantly influenced the shear behavior. Increases in *S* (decreases in *N*) maximize interface shear resistance when the elements are largely spaced and the shear resistance decreases as *S* decreases (or *N* increases), whereas increases in *N* caused a decrease in mobilized shear resistance. The results also showed that the element to height ratio (*S/h*) also significantly influenced the shear resistance. Increase in *S/h* ratio resulted in an increase in shear resistances at the interface between textured surfaces and test sands. The effect of particle size of test sand on shear resistance diminishes as *N* increases or *S/h* decreases (i.e., closely spaced elements).

## Data Availability

All data generated or analyzed during this study are included in this published article.
